# Tuberculous pleural effusion prediction using ant colony optimizer with grade-based search assisted support vector machine

**DOI:** 10.3389/fninf.2022.1078685

**Published:** 2022-12-19

**Authors:** Chengye Li, Lingxian Hou, Jingye Pan, Huiling Chen, Xueding Cai, Guoxi Liang

**Affiliations:** ^1^Department of Pulmonary and Critical Care Medicine, The First Affiliated Hospital of Wenzhou Medical University, Wenzhou, China; ^2^Department of Rehabilitation, Wenzhou Hospital of Integrated Traditional Chinese and Western Medicine, Wenzhou, China; ^3^Key Laboratory of Intelligent Treatment and Life Support for Critical Diseases of Zhejiang Province, Wenzhou, Zhejiang, China; ^4^Collaborative Innovation Center for Intelligence Medical Education, Wenzhou, Zhejiang, China; ^5^Zhejiang Engineering Research Center for Hospital Emergency and Process Digitization, Wenzhou, Zhejiang, China; ^6^Department of Intensive Care Unit, The First Affiliated Hospital of Wenzhou Medical University, Wenzhou, Zhejiang, China; ^7^College of Computer Science and Artificial Intelligence, Wenzhou University, Wenzhou, Zhejiang, China; ^8^Department of Pulmonary and Critical Care Medicine, The First Affiliated Hospital of Wenzhou Medical University, Wenzhou, Zhejiang, China; ^9^Department of Information Technology, Wenzhou Polytechnic, Wenzhou, China

**Keywords:** tuberculous pleural effusion, swarm intelligence, machine learning, feature selection, support vector machine

## Abstract

**Introduction:**

Although tuberculous pleural effusion (TBPE) is simply an inflammatory response of the pleura caused by tuberculosis infection, it can lead to pleural adhesions and cause sequelae of pleural thickening, which may severely affect the mobility of the chest cavity.

**Methods:**

In this study, we propose bGACO-SVM, a model with good diagnostic power, for the adjunctive diagnosis of TBPE. The model is based on an enhanced continuous ant colony optimization (ACOR) with grade-based search technique (GACO) and support vector machine (SVM) for wrapped feature selection. In GACO, grade-based search greatly improves the convergence performance of the algorithm and the ability to avoid getting trapped in local optimization, which improves the classification capability of bGACO-SVM.

**Results:**

To test the performance of GACO, this work conducts comparative experiments between GACO and nine basic algorithms and nine state-of-the-art variants as well. Although the proposed GACO does not offer much advantage in terms of time complexity, the experimental results strongly demonstrate the core advantages of GACO. The accuracy of bGACO-predictive SVM was evaluated using existing datasets from the UCI and TBPE datasets.

**Discussion:**

In the TBPE dataset trial, 147 TBPE patients were evaluated using the created bGACO-SVM model, showing that the bGACO-SVM method is an effective technique for accurately predicting TBPE.

## 1 Introduction

According to WHO Global tuberculosis report 2016, there was an approximately 10.4 million new cases of TB and 1.4 million deaths caused by TB. Six countries with the most severe cases of TB were China, South Africa, India, Indonesia, Nigeria, and Pakistan (Pan, 2012). Besides, the most common pulmonary tuberculosis, there is tuberculous pleurisy and so on. One of the most common causes of pleural effusion is tuberculous pleurisy. Tuberculous pleurisy is often manifested as non-productive cough, fever, chest pain, dyspnea, etc. Those patients suspected of tuberculous pleurisy need rapid and accurate diagnosis and prompt treatment, otherwise it will develop into tuberculous empyema, thoracic malformations and other serious consequences, and even death.

Diagnosis of tuberculous pleurisy relies on sputum, pleural effusion, pleural biopsy specimen culture, or molecular linear probe assay to detect Mycobacterium tuberculosis (World Health Organization, [WHO], 2010). The patient often presents with a non-productive cough and less bacteria in pleural fluid so the culture has low sensitivity. With long incubation, timely diagnosis is almost impossible (Gopi et al., 2007; von Groote-Bidlingmaier et al., 2013). Pleural biopsy for invasive examination includes blind biopsy and Thoracoscopic pleural biopsy. Blind biopsy has low sensitivity and it can easily cause pneumothorax. Thoracoscopic examination requires higher infrastructure and technical skills, difficult to access in regions with poor economy and higher incidence of TB. Other common examinations, such as acid-fast Mycobacterium tuberculosis smear, Xert MTB/RIF detection on samples, IFN-r assay, pleural fluid cell count, and biochemical detection etc. exist but have moderate sensitivity, low specificity, and high cost (Seibert et al., 1991; Conde et al., 2003; Jiang et al., 2007; Udwadia and Sen, 2010; Porcel et al., 2013; Lee et al., 2014). In the areas with high prevalence of tuberculosis, the most frequent cases of tuberculous pleurisy were inferred from a predominantly lymphocytic exudate combined with high adenosine deaminase (ADA), which is comparatively better but there was still a false negative or false positive, especially in early tuberculous pleurisy (Lee et al., 2014).

Others diagnose tuberculous pleurisy using diagnostic models. In addition, artificial general intelligence methods (Yang S. et al., 2021; Yang et al., 2022a,b,c) are critical studies in the field of brain-inspired intelligence to realize high-level intelligence, high accuracy, high robustness, and low power consumption in comparison with the state-of-the-art artificial intelligence works. Seixas et al. (2013) employ artificial neural networks (ANN), the non-invasive prediction model established for the pleural effusion smear, culture, ADA, serology, and nucleic acid amplification (NAA) test results in HIV-infected patients, and the detection accuracy was > 90%. Shu et al. (2015) use logistic regression analysis in patients with predominantly lymphocytic pleural effusion to analyze the inflammatory cytokines, anti-inflammatory cytokines and T lymphocyte effector molecules, including total protein, cell and classification counts, culture of bacteria, lactate dehydrogenase (LDH), fungi, and chemokines [monocyte chemo-attractant protein (MCP)-1, cytology, cytokines interleukin (IL)-1β, IL-2, IL-6, IL-10, IL-12, IL-13, TNF-α, and IFN-γ, mycobacteria, macrophage inflammatory protein (MIP)-1α, regulated on activation, normal T cell expressed and secreted, and IP-10], soluble tumor necrosis factor receptor TNF-sR1 and TNF-sR2, vascular endothelial growth factor (VEGF) and establish the diagnostic model. The results showed ADA ≥ 40 IU/mL, IFN-γ ≥ 75 pg/mL, DcR3 ≥ 9.3 ng/mL, and soluble tumor necrosis factor receptor 1 (TNF-sR1) ≥ 3.2 ng/mL which were independent factors associated with tuberculous pleurisy. Based on the prediction probability of four predictors, the area under ROC curve was 0.920, and the specificity and sensitivity were 86.7 and 82.9%, respectively (Shu et al., 2015). Klimiuk et al. (2015) used ROC analysis and multiple regression analysis to construct the prediction model of tuberculous pleurisy. According to the patient’s clinical data and multiple pleural effusion biomarkers (ADA, IFN-γ, IL-2, IL-2sRα, IL-12p40, IL-18, IL-23, IP-10, Fas-ligand, MDC, and TNF-α), the diagnostic accuracy (AUC) of tuberculous pleurisy was higher than 0.95 (Klimiuk et al., 2015). Demirer used unrestricted logistic regression method to distinguish tuberculous pleural effusion (TBPE) from the non-TBPE, which only relies on the general condition of a patient with pleural effusion, imaging studies, results of pleural effusion routine test. Results showed that specificity and sensitivity were 83.0 and 60.5% and AUC was 0.719 when only the age was less than 47 and the pleural fluid ADA more than 35 or pleural fluid protein serum protein ratio was higher than 0.71 (Demirer et al., 2012). Some of these models need to detect unusual molecular markers, which are expensive. Since some models rely on pleural fluid detection, it’s difficult to use the model when the fluid is difficult to obtain, such as inadequate fluid, a scapular posterior pleural effusion, patient has severe pleural reaction, and so on.

In computer-aided diagnosis technology, machine learning methods play an important role, and the classification and prediction ability of support vector machine (SVM) has been fully validated not only in the medical field, but also by many scholars in various other fields, and many researches on SVM have been conducted for this purpose. For example, in medical diagnosis fields, Zhou et al. (2022) developed a SVM-based discriminating model to extract a set of candidate biomarkers for malignant brain gliomas. Gao et al. (2022) proposed a novel kernel-free ν-fuzzy reduced kernel-free quadratic surface SVM model and applied it to Alzheimer’s Disease prodromal detection. Badr et al. (2022) applied a recent gray wolf optimizer to improve the performance of SVM for breast cancer diagnosis with efficient scaling techniques. Vidya and Gait (2021) presented a gait classification based decision support system using multi-class SVM to assist the clinicians to diagnose the Parkinson’s disease and rate the severity level. Chen B. et al. (2021) implemented an extended kalman filter with SVM for automated brain tumor detection. Viloria et al. (2020) applied a SVM to predict the diagnosis of diabetes according to the above factors in patients. Akinnuwesi et al. (2020) adopted the hybrid of principal component analysis (PCA) and SVM to establish breast cancer risk assessment and early diagnosis model which can accurately establish the early diagnosis model of breast cancer. In other fields, Devi Thangavel et al. (2023) used fuzzy logic and multi-class SVM techniques to properly set and monitor model parameters such as suitable temperature, humidity, and soil moisture for greenhouse farms located near Modaculic Erosion. Zhao Z. et al. (2022) proposed a new residual-type combined Gray Model-Least Squares Support Vector Machine (LSSVM) forecasting model by extracting the load characteristics of components. Zhang J. et al. (2022) proposed a two-stage intelligent fault diagnosis methodology for rotating machinery based on optimized support vector data description to optimize SVM. Wang S. et al. (2022) proposed a novel optimization method named synergy adaptive moving window algorithm based on the immune SVM to select wavelength variables or preprocessing methods in near-infrared spectroscopy. Pathivada and Vedagiri (2022) evaluated the dilemma frontier under mixed traffic levels using SVM, contributing to better understand the dilemma frontier in developing countries under mixed traffic conditions, such as India.

Based on the above research on SVM, it is easy to find that SVM has powerful classification and prediction ability, and has been widely used in many fields, among which the contribution in the field of medical diagnosis is indelible. In previous study, we used the SVM method in the machine learning algorithm to diagnose TBPEs. The general clinical condition, blood biochemical parameters and routine pleural fluid analysis had very good diagnostic accuracy of 94.3%, sensitivity and specificity were 93.6 and 94.1%. We envisage the ability to diagnose tuberculous pleural fluid that is difficult to obtain by pleural effusion by detecting the blood of our patients. In the end we employed the bGACO-SVM method, a swarm intelligence algorithm to establish the diagnosis of tuberculous pleurisy model, only based on patient general condition and routine blood test results. The diagnostic accuracy of ACC reached 96.57%, Matthew correlation coefficient (MCC) was 0.9366, with F-measure and specificity 96.65 and 96.91%, respectively.

In this study, the ACOR is first reviewed, and then an in-depth study is conducted to propose an ant colony optimizer with a grade-based search, called grade-based search technique (GACO), focusing on the aspects of ACOR in terms of the convergence accuracy and avoiding falling into local optimum. In GACO, a grade-based search mechanism with strong convergence properties is mainly introduced into the original ACOR, which effectively improves the convergence accuracy of GACO and further enhances the ability to avoid falling into local optima. In order to prove the performance of GACO, a comparative simulation was conducted between GACO and nine basic algorithms and nine similar variant optimization algorithms, and the Wilcoxon signed-rank test (García et al., 2010) and Friedman test (Derrac et al., 2011) were utilized to evaluate the experimental results, which effectively proved that GACO has a strong convergence ability and the ability to avoid being trapped in a local optimum. Finally, to achieve diagnostic prediction of TBPE, the GACO was first transformed into a binary version, called bGACO, and subsequently an SVM classifier with both feature selection functions, called bGACO-SVM, was proposed based on bGACO. For the proposed bGACO-SVM, it is not only validated on some very common public datasets, but also applied to the TBPE prediction problem. bGACO-SVM is effectively demonstrated to have strong classification prediction capability and can be successfully used for TBPE diagnosis and prediction through experimental simulation results.

To summarize, the important contributions and innovations of this paper are as follows.

➢ A novel swarm intelligence optimization algorithm, called GACO, is proposed by introducing a grade-based search method to ACO.
➢ The grade-based search greatly improves the convergence performance of GACO and the ability to avoid getting trapped in local optimization, thus improving the classification capability of bGACO-SVM.
➢ Both GACO and nine basic algorithms and nine state-of-the-art variants are compared with each other, providing strong evidence of GACO’s core strengths.
➢ The prediction accuracy of bGACO-SVM was evaluated using existing datasets from the UCI and TBPE datasets, showing that the bGACO-SVM method can accurately predict TBPE.

The follow-up of the paper is organized as follows. Section II describes the data acquisition of TBPE and other related contents. The review of ACOR and the proposed GACO are given in Section 3. The construction process of bGACO-SVM model is given in Section 4. Section 5 first validates the performance of GACO by way of experiments and then simulates the diagnosis prediction of TBPE using bGACO-SVM. Section 6 discusses all the research work in this paper. Section 7 summarizes the whole paper and discusses future works.

## 2 Data analysis

### 2.1 Patient information

The research was prospectively conducted on the 147 patients with pleural effusion that were admitted to the First Affiliated Hospital Wenzhou Medical University from October 2015 to May 2016, with an age greater than 15 years and no HIV infection. This research was carried out in accordance with the declaration of Helsinki and approved by the Medical Ethics Committee of The First Affiliated Hospital Wenzhou Medical University.

The results of growth of mycobacterium tuberculosis in biopsy specimens or pleural fluid culture were utilized as the gold standard for the diagnosis of tuberculous pleuritis (World Health Organization, [WHO], 2010). The pathogenesis of pleural effusion was determined by means of thoracic puncture, closed pleural blindness, internal medicine thoracoscopy, bone marrow puncture, blood test, pleural effusion and pleural tissue culture, and pathological examination. Results 73 patients were diagnosed with tuberculous pleurisy. There are 67 patients with non- TBPE, including transudate (Light et al., 1972) (postrenal transplantation, heart failure), malignant tumor, infectious disease, connective tissue disease, and hematopathy. 7 patients were not included in the research because the cause could not be determined see Table 1.

**TABLE 1:** The features at diagnosis of PE.

Causes	n	%	Males/females	Age, years^a^
Tuberculosis	73	49.70%	54/19	41.8 ± 19.9
Neoplastic	43	29.30%	21/22	66.2 ± 11.4
Infectious	18	l2.2%	16/2	57.8 ± 17.9
Lymphoma	2	1.40%	2/0	
Renal transplantation	1	0.70%	1/0	
Heart failure	1	0.70%	1/0	
Multiple myeloma	1	0.70%	0/1	
Undifferentiated connective tissue disease	1	0.70%	1/0	
Unclassified	7	4.7i%	5/2	50.3 ± 17.2
Total	147	100%	101/46	

^a^Mean ± standard deviation.

### 2.2 Statistical analysis

With medical history and physical examination, general clinical data were collected including body mass index, sex, age, body temperature, and diabetes status. The fasting venous blood was collected from all subjects using a vacuum blood collection vessel (Becton Dickinson, Medical Devices Co., Ltd., NC, USA). The Hospital Inspection Center used Japan’s sysmex XE—2100 automatic blood cell analyzer (Sysemx Corporation, Kobe, Japan) to conduct 24 routine blood indices. See Table 2 for a detailed description.

**TABLE 2:** The whole features utilized in this research and their descriptions.

Item	Brief description
ID1	High (H)
ID2	Weight (W)
ID3	Diabetes (DM)
ID4	Temperature (Tem)
ID5	Sex
ID6	Age
ID7	White blood cell (WBC)
ID8	Percentage of neutrophils (PN)
ID9	Percentage of eosinophils (PE)
ID10	Percentage of basophils (PB)
ID11	Percentage of monocyte (PM)
ID12	Percentage of lymphocytes (PL)
ID13	Absolute value of eosinophils (AVE)
ID14	Absolute value of neutrophils (AVN)
ID15	Absolute value of monocyte AVM
ID16	Absolute value of lymphocytes (AVL)
ID17	Absolute value of basophils (AVB)
ID18	Red blood cell (RBC)
ID19	Hemoglobin (HB)
ID20	Hematokrit (HCT)
ID21	Mean corpuscular volume (MCV)
ID22	Mean corpuscular hemoglobin (MCH)
ID23	Mean corpuscular hemoglobin concentration (MCHC)
ID24	Red cell volume distribution width (RBCDW)
ID25	Red cell volume distribution SD value (RBCVDSD)
ID26	Blood platelet (PLT)
ID27	Thrombocytocrit (THR)
ID28	Mean platelet volume (MPL)
ID29	Platelet distribution width SD value (PDWSDV)
ID30	Platelet large cell ratio (P-LCR)

SPSS 19 was used for statistical analysis. Using analysis of ANOVA and chi square test, the general clinical data and blood routine test of both patients of TBPE and non-TBPE were analyzed to find out the statistical differences. In all analyses, a *p*-value of less than 0.05 (5% significant level) was considered significant. The detailed statistics are shown in Tables 3, 4.

**TABLE 3:** The clinical features of TPE patients and Non-TPE patients.

Feature	TPE (*n* = 73)	NTPE (*n* = 67)	*P*-value	χ^2^-value
Sex Male/Female	54/19	41/26	0.106	2.615
DM Yes/No	5/68	6/61	0.644	0.214

DM, diabetes.

**TABLE 4:** Biochemical and clinical parameters in TPE patient and Non-TPE patient.

Index	TPE (*n* = 73)	Min	Max	SD	NTPE (*n* = 67)	Min	Max	SD	*p*-value
	Mean				Mean				
H (cm)	165.84	150.00	182.00	7.06	163.99	150.00	176.00	6.04	0.1
W (kg)	58.99	38.00	85.00	9.44	60.00	40.00	80.00	8.49	0.508
Tem (°C)	38.51	37	40	0.78	37.64	36.5	39.9	0.66	<0.001
AGE (years)	41.85	15	81	19.87	63.19	25	88	14.63	<0.001
WBC (10^9/L)	7	3.12	16.83	2.31	8.58	2.86	22.82	3.76	0.003
PN (100%)	0.6771	0.49	0.910.06	0.0864	0.6756	0.11	0.9	0.134	0.935
PE (100%)	0.0156	0	0.01	0.013	0.0265	0.06	0.19	0.0312	0.007
PB (100%)	0.0021	0	0.86	0.0017	0.0024	0	0.01	0.0024	0.378
PM (100%)	0.1355	0.04	0.39	0.0922	0.0869	0.01	0.21	0.0336	<0.001
PL (100%)	0.1802	0.04	0.41	0.069	0.2119	0.04	0.85	0.1258	0.063
AVE (10^9/L)	0.1	0	12.87	0.08	0.31	0	4.42	0.77	0.02
AVN (10^9/L)	4.81	2.05	2.04	2	5.923	0.13	20.5	3.81	0.032
AVM (10^9/L)	0.85	0.15	2.76	0.35	0.7	0.1	2	0.37	0.017
AVL (10^9/L)	1.2	0.33	0.09	0.47	1.6	0	8.6	1.11	0.006
AVB (10^9/L)	0.0145	0	5.52	0.0135	0.0193	0	0.1	0.0225	0.13
RBC (10^12/L)	4.51	2.71	165	0.59	4.21	2.65	5.75	0.72	0.007
HB (pg)	130.93	84	0.49	18.09	125.79	76	178	19.74	0.11
HCT (L/L)	0.3892	0.26	98.8	0.0476	0.3726	0.23	0.52	0.0577	0.064
MCV (fl)	86.48	62.1	32.7	4.81	89.23	71.3	103.4	5.58	0.002
MCH (pg)	29.05	18.5	360	2	30.1	21.5	34.6	1.97	0.002
MCHC (g/L)	335.88	297	16.9	12.49	337.78	302.11	372	13.95	0.397
RBCDW (%)	12.84	11.5	54.1	0.99	13.33	11.8	17	1.03	0.004
RBCVDSD (fl)	40.42	30.4	601	3.71	42.92	35.8	60.2	4.09	<0.001
PLT (10^9/L)	365.15	169	0.57	91.32	293.01	67	670	135.19	0.001
THR (L/L)	0.3474	0.19	12.9	0.083	0.2997	0.1	0.64	0.123	0.008
MPL (fl)	9.73	1.5	17.7	1.38	10.31	6.6	13.4	1.13	0.008
PDWSDV (fl)	11.08	7.7	49.5	1.85	12.46	1.2	19.1	2.89	0.001
P-LCR (%)	23.47	9.6	22	7.78	28.4	13.1	52.7	8.4	0.001

## 3 The proposed GACO

In this subsection, a review of ACOR is presented, followed by an introduction of the Grade-based search strategy, and finally a detailed description of the process and significance of GACO proposal.

### 3.1 An overview of ACOR

In recent years, swarm intelligence optimization algorithms have been widely applied to various fields, and therefore have been developed rapidly, and many excellent algorithms have sprung up. For example, there are some basic optimization algorithms including gray wolf optimization (GWO) (Mirjalili et al., 2014), Harris hawks optimization (HHO) (Heidari et al., 2019), moth-flame optimization (MFO) (Mirjalili, 2015), particle swarm optimization (PSO) (Kennedy and Eberhart, 1995), whale optimizer (WOA) (Mirjalili and Lewis, 2016), Runge Kutta optimizer (RUN) (Ahmadianfar et al., 2021), hunger games search (HGS) (Yang Y. et al., 2021), weighted mean of vectors (INFO) (Ahmadianfar et al., 2022), colony predation algorithm (CPA) (Tu et al., 2021), slime mold algorithm (SMA) (Li S. et al., 2020), JAYA optimization algorithm (Rao, 2016), firefly algorithm (FA) (Yang, 2009), stochastic fractal search (SFS) (Salimi, 2015), and ant colony optimization for continuous domains (ACOR) (Socha and Dorigo, 2008). In addition, there are some advanced variant algorithms, such as fruit fly optimizer with multi-population outpost mechanism (MOFOA) (Chen et al., 2020), bat algorithm based on collaborative and dynamic learning of opposite population (CDLOBA) (Yong et al., 2018), hybridizing gray wolf optimization (HGWO) (Zhu et al., 2015), opposition-based sine cosine algorithm (OBSCA) (Abd Elaziz et al., 2017), Moth-flame optimizer with sine cosine (SMFO) (Chen C. et al., 2021), Cauchy and Gaussian sine cosine algorithm (CGSCA) (Kumar et al., 2017), modified SCA (m_SCA) (Qu et al., 2018), double adaptive random spare reinforced whale optimization algorithm (RDWOA) (Chen et al., 2019), and associative learning-based exploratory whale optimizer (BMWOA) (Heidari et al., 2020). Furthermore, they are already making their impact in many fields, such as train scheduling (Song et al., 2023), image segmentation (Hussien et al., 2022; Yu et al., 2022), feature selection (Liu Y. et al., 2022), complex optimization problem (Deng et al., 2022a), bankruptcy prediction (Zhang et al., 2021), gate resource allocation (Deng et al., 2020; Wu D. et al., 2020), multi-objective problem (Hua et al., 2021; Deng et al., 2022d), expensive optimization problems (Li J.-Y. et al., 2020; Wu S.-H. et al., 2021), robust optimization (He et al., 2019, 2020), airport taxiway planning (Deng et al., 2022c), scheduling problems (Gao et al., 2020; Han et al., 2021; Wang G.-G. et al., 2022), medical diagnosis (Chen et al., 2016; Wang et al., 2017), and resource allocation (Deng et al., 2022b).

Besides, no single algorithm is one-size-fits-all and can solve every issue, as stated in the notion of no free lunch (Wolpert and Macready, 1997). Therefore, we designed a novel feature selection method based on continuous ant colony optimization (ACOR) (Socha and Dorigo, 2008). The proposed feature selection method has also been successfully applied to TBPE prediction. ACOR is proposed firstly by Socha and Dorigo (2008), which has been applied to a wide variety of realistic scenarios with relatively unexpected success, such as image segmentation (Liu et al., 2021; Zhao et al., 2021a,b), engineering optimization design (Chen and Wang, 2014; Zhao D. et al., 2022), path planning (Liu J. et al., 2022), image registration (Wu et al., 2019), energy optimization (Fetanat and Khorasaninejad, 2015), and maintenance scheduling problem (Fetanat and Shafipour, 2011). As far as we know, few studies have used this approach to address this TBPE prediction problem.

**FIGURE 1:**
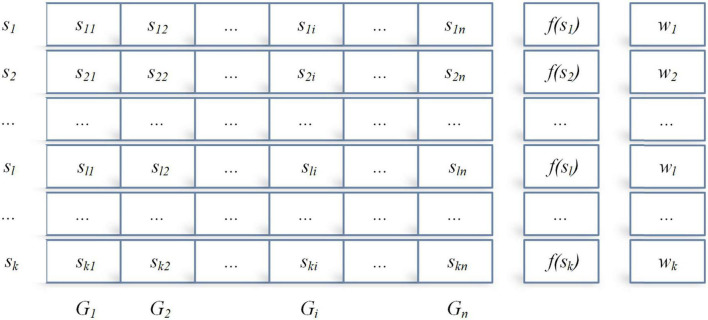
The archive of solutions in AOCR.

In the design and implementation of ACOR, the core idea is the archive theory shown in Figure 1, which stores the ant individuals, the individual fitness values, and the individual weight.

where *s*_*l*_ = {*s*_*l*1_,…,*s*_*ln*_} denotes an ant individual *l*, and *f*(*s*_*l*_) and *w*_*l*_ denote the corresponding fitness value and weight value of individual *l*, respectively. Between *f*(*s*_*l*_) and *w*_*l*_, if *f*(*s*_1_)≤…*f*(*s*_*l*_)≤…*f*(*s*_*k*_), then the foot *w*_1_≥…*w*_*l*_≥…*w*_*k*_.

The continuous probability density function is the core of ACOR. Among the common probability density functions, the Gaussian function, although easy to sample, has only one peak for a single Gaussian function, and when there are two or more peaks, the Gaussian function cannot be handled well. Therefore, ACOR uses the Gaussian kernel function *G*_*i*_(*x*) as the probability density function, and it is actually a weighted sum of several one-dimensional Gaussian functions, as shown in Eq. (1).


(1) $$G_{i}\,\left(s_{l\,i}\right)=\sum_{l=1}^{k}w_{l}\,g_{l\,i}\,\left(x\right)=\sum_{l=1}^{k}w_{l}\,\frac{1}{\sigma_{l\,i}\,\sqrt{2\,\pi }}\,e^{-\frac{{\left(s_{l\,i}-\mu_{l\,i}\right)}^{2}}{2\,\sigma_{l\,i}^{2}}}$$

where *k* is the number of individual ants composing the archive bag, $\left\{\mu_{1}^{i},\ldots ,\mu_{k}^{i}\right\}$ is the mean vector in the Gaussian function *G*_*i*_(*s*_*li*_), and $\left\{\sigma_{1}^{i},\ldots ,\sigma_{k}^{i}\right\}$ is the standard deviation vector related to *G*_*i*_(*s*_*li*_) in a single dimension.

In the actual implementation, firstly, the guide individual *s*_*l*_ is selected by roulette based on the weight *w*. The greater the weight of the guide individual, the higher the chance of being selected, and then new ant individuals are generated by exploring around the guide individual *s*_*l*_. The exploration process mainly relies on the constructed Gaussian function. Finally, the m ant individuals generated and the k individuals in the archive are merged, and the *m* poorer individuals are removed from the merged archive, which is also the process of pheromone update of ant individuals.

### 3.2 Grade-based search strategy

The grade-based search strategy (GS) is a mechanism abstracted from GWO, where the core is mainly to simulate the hierarchy of gray wolves. As a swarm intelligence optimization algorithm proposed by Mirjalili et al. (2014) in 2014, GWO is characterized by few parameters, strong convergence performance and easy implementation. Therefore, based on the inspiration of the above ideas, GS is introduced into this paper to improve the convergence performance of ACOR.

In the GS, ant individuals are also classified into four classes: α, β, δ, and ω. There are the α, β, and δ leaders in the population, representing the current optimal candidate solution, second best solution, and third best solution, independently. Other individual ants are called ω, which follow α, β, and δ to search for food. Therefore, ant individuals searching for food can be represented by the following mathematical model.


(2) D⇀=|C⇀⋅Xf⇀f(t)-X⇀(t)|

(3) X⇀(t+1)=Xf⇀f(t)-A⇀⋅D⇀

where *t* represents the number of current iterations, $\vec{A}$ and $\vec{C}$ are coefficient vectors, ${\vec{X}}_{f}$ indicates the position vector of food, and $\vec{X}$ is the location vector of ant individual. $\vec{A}$ and $\vec{C}$ can be obtained according to the following Eqs. 4, 5.


(4) A⇀=2a⇀⋅r1⇀1-a⇀

(5) C⇀=2⋅r2⇀2

where $\vec{a}$ decreases linearly from 2 to 0 as the iteration. ${\vec{r}}_{1}$ and ${\vec{r}}_{2}$ are all random number vectors between [0, 1].

Furthermore, the ant individual takes the current location as the optimal food position when | A | < 1. In contrast, the ant moved away from the food when | A | > 1 and searched for other food. The central position of α, β, and δ is taken as the optimal solution since the position of the optimal food is unknown. Other ant individuals update their positions according to these three optimal ant individuals with the following Eqs. (6–8).


(6) $$\left\{ \begin{array}{c} \vec{D_{\alpha }}=\left|\vec{C_{a}}\cdot \vec{X_{\alpha }}\,\left(t\right)-\vec{X_{i}}\,\left(t\right)\right|\\ \vec{D_{\beta }}=\left|\vec{C_{b}}\cdot \vec{X_{\beta }}\,\left(t\right)-\vec{X_{i}}\,\left(t\right)\right|\\ \vec{D_{\delta }}=\left|\vec{C_{c}}\cdot \vec{X_{\delta }}\,\left(t\right)-\vec{X_{i}}\,\left(t\right)\right| \end{array}\right.$$

(7) $$\left\{ \begin{array}{c} \vec{X_{1}}=\vec{X_{\alpha }}-\vec{A_{a}}\cdot \vec{D_{\alpha }}\\ \vec{X_{2}}=\vec{X_{\beta }}-\vec{A_{b}}\cdot \vec{D_{\beta }}\\ \vec{X_{3}}=\vec{X_{\delta }}-\vec{A_{c}}\cdot \vec{D_{\delta }} \end{array}\right.$$

(8) $$\vec{X_{i}}\,\left(t+1\right)=\frac{\vec{X_{1}}+\vec{X_{2}}+\vec{X_{3}}}{3}$$

### 3.3 The proposed GACO

By reviewing the key elements of ACOR in section “3.1 An overview of ACOR,” it can be seen that ACOR relies mainly on the constant updating of individuals in the archive to obtain the optimal solution. Therefore, since each iteration removes the inferior solutions and retains the superior ones, this allows the ant individuals to continuously move closer to the optimal ones, but this also leads to the same problem of reduced diversity of ant individuals, poor convergence and easy to fall into local optimum. To make up for these shortcomings as much as possible, this paper introduces the GS strategy in ACOR to form a new continuous ant colony optimizer, called GACO. The GS strategy mainly simulates the wolf pack hierarchy and group hunting behavior in GWO, and it mainly acts on the population as a whole after merging the ant individuals in the archive and the newly generated ant individuals. Since the first half of wolf foraging emphasizes more on the global performance in optimization and the second half emphasizes more on the local performance, the introduction of GS strategy then makes ACOR have better convergence performance and stronger ability to jump out of the local optimum. See Algorithm 1 for the pseudo-code of the proposed GACO.

**Algorithm 1:** Pseudo-code of GACO.

**Input:** The fitness function *f(s)*, maximum evaluation number (*MaxFEs*), the parameter *q*, archive size(*k*), population size (*m*), dimension (*n*), pheromone evaporation rate(ξ) **Output:** The best ant (*bestAnt*) Initialize the parameters *MaxFEs*,*q*,*k*,*m*,ξ; Initialize the population of *k* ants in archive; *s* = ∅; **For** *l* = 1 to *k* *s*_*l*_ = *rand*(*UL*); *s* = *s*∪*s*_*l*_; *f*_*l*_ = *f*(*s*_*l*_); **End For** *s* = *sorting*(*s*); $w_{l}=\frac{1}{q\,k\,\sqrt{2\,\pi }}\,e^{-\frac{{\left(l-1\right)}^{2}}{2\,q^{2}\,k^{2}}}$; $p_{l}=\frac{w_{l}}{\sum_{r=1}^{k}w_{r}}$; *BestAnt* = *x*_1_; **While** (*FEs* ≤ *MaxFEs*) Generate the empty population of *m* ants; **For** *i* = 1 to *m* Choose a solution *s*_*c*_ according to probability *p*_*c*_ where *c* ∈ [1,*k*]; **For** *j* = 1 to *n* μ_*ij*_ = *s*_*cj*_; $\sigma_{i\,j}=\xi \,\sum_{e=1}^{k}\frac{\left|s_{e\,j}-s_{c\,j}\right|}{k-1}$; *x*_*ij*_ = 𝒩(μ_*ij*_, σ_*ij*_); **End For** *f*_*k* + *i*_ = *f*(*x*_*i*_); *x* = *s* ∪ *x*_*i*_; **End For** *x* = *sorting*(*x*); **For** *l* = 1 to *k* + *m* Form $x_{l}^{\prime }$ by simulating GS strategy; $f_{l}^{\prime }=f\,\left(x_{l}^{\prime }\right)$; **If** $f_{l}<f_{l}^{\prime }$ $x_{l}=x_{l}^{\prime }$; **End If** **End For** *x* = *sorting*(*x*); *x* = *x* − *s*_*m*_; *bestAnt* = *x*_1_; **End While** **Return** *bestAnt*

## 4 The proposed bGACO-sVM model

### 4.1 Binary transformation method

Feature selection technique is regarded as a binary optimization problem. In order to solve the feature selection issue, an enhanced binary version based on the GACO algorithm is put forward. In this study, the solution is represented as a *d* dimension vector, where *d* is the number of attributes of the dataset. The original update method of the GACO algorithm is useless when handling binary optimization issues because these solutions do not only have the “0” and “1” values. In order to settle the problem, it discretizes the position vector of individual ants into a binary value. The updated formula is defined as following.


(9) $$X_{d}\,\left(t+1\right)=\left\{ \begin{array}{c} 1,s\,i\,g\,m\,o\,i\,d\,\left(X_{d}\,\left(t\right)\right)\geq r\,a\,n\,d\\ 0,o\,t\,h\,e\,r\,w\,i\,s\,e \end{array}\right.$$

where rand denotes a random number obeying a uniform distribution between [0,1]⋅*X*_*d*_(*t* + 1) is an iteration of t using binary position update. the specific expression of sigmoid is shown below.


(10) $$s\,i\,g\,m\,o\,i\,d\,\left(x\right)=\frac{1}{1+e^{-10\,\left(x-0.5\right)}}$$

In this way, the original continuous problem is transformed into a discrete problem. In addition, in order to further evaluate the importance of the selected features, machine learning methods are added to the evaluation of the fitness values to further select the most effective features.

### 4.2 Support vector machine

SVM has been applied to many practical issues such as breast cancer diagnosis (Huang et al., 2019), TBPE diagnosis (Li et al., 2018), analysis of patients with paraquat poisoning (Hu et al., 2017), prognosis of patients with paraquat poisoning (Chen et al., 2017), prediction of electricity price (Weron, 2014), prediction of electricity spot-prices (Cincotti et al., 2014), and prediction of Parkinson’s disease (Cai et al., 2017). The mechanism of SVM is to find an optimal plane that can maximally separate different data. The support-vector is the data point closest to the boundary. In data processing, SVM is often used as a supervised learning method to decide the optimal hyperplane which distinguish positive and negative samples accurately. The hyperplane is defined as follows, giving the dataset G = (*x*_*i*_,*y*_*i*_),*i* = 1,…,N,*x* ∈ *R^d^*,*y* ∈ {±1}.


(11) $$\text{g}\,\left(x\right)=\omega^{T}\,x+b$$

Minimization in terms of geometric comprehension of the hyperplane equals to the maximization of geometric spacing equals minimization. In the presence of a small number of outliers, the “soft interval” idea is added, and the slack variable ξ_*i*_ > 0 is utilized. The discipline factor c represents the ability to accept outliers and is one of the main factors affecting the effectiveness of SVM classification. The standard SVM model is shown in the figure below.


(12) $$\left\{ \begin{array}{c} \min\left(\omega \right)=\frac{1}{2}\,{∥\omega ∥}^{2}+c\,\sum_{i=1}^{N}\xi_{i}^{2}\\ s.t\,y_{i}\,\left(\omega^{\text{T}}\,x_{i}+b\right)\geq 1-\xi_{i},i=1,2,\ldots ,N \end{array}\right.$$

where ω denotes the weight of inertia, *b* represents a constant.

This method transforms low-dimensional data i into high-dimensional data and combines the multivariate linear techniques to partition the optimal classification surface. At the same time, SVM changes the set of linearly indivisible samples Φ:*R^d^*→*H* non-linearly. In order to ensure that the computational outcomes of the high-dimensional part are the same as the low-dimensional part, an appropriate kernel function k(*x*_*i*_,*x*_*j*_) is constructed, with α_*i*_ indicating the Lagrange multiplier and Eq. (3) being transformed as follows:


(13) $$\left\{ \begin{array}{c} Q\,\left(\alpha \right)=\frac{1}{2}\,\sum_{i=1}^{N}\alpha_{i}\,\alpha_{j}\,y_{i}\,y_{j}\,k\,\left(x_{i},x_{j}\right)-\sum_{i=1}^{N}\alpha_{i}\\ s.t\,\sum_{i=1}^{N}a_{i}\,y_{i}=0,0\leq a_{i}\leq C,i=1,2,\,\ldots ,N \end{array}\right.$$

In this paper, the generalized radial basis kernel function is adopted, and its expression is as follows.


(14) $$k\,\left(x,y\right)=e^{-\gamma \,∥x_{i}-x_{j}∥}$$

where γ is another factor that is very important for the classification performance of SVM and denotes a kernel parameter that specifies the interaction width of the kernel function.

### 4.3 The proposed bGACO-SVM model

This section proposes a novel and efficient model based on the bGACO and the SVM for feature selection experiments, named bGACO-SVM model. The model is mainly used to select key features from the dataset. The fitness of the selected feature subset for each individual ant is evaluated during the feature selection process. The specific fitness values are calculated as follows.


(15) $$F\,i\,t\,n\,e\,s\,s=\alpha \cdot e\,r\,r\,o\,r+\beta \cdot \frac{\left|R\right|}{\left|D\right|}$$

where *er* denotes the SVM classification error rate, |*D*| represents the number of features in the dataset. |*R*| denotes the number of features of the selected feature subset. α is a weight that measure the importance of the classification error rate and β is a weight that measure the length of the selected features, respectively. In our research, α = 0.99 and β = 0.01 are set, and both are commonly used in many works.

**FIGURE 2:**
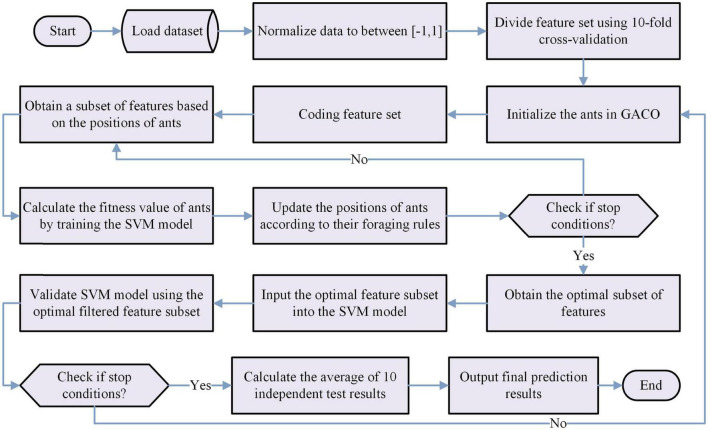
Flow chart of the bGACO-SVM model.

In summary, we can obtain the bGACO-SVM model by combining the proposed bGACO with the SVM in this paper, and its workflow is shown in Figure 2.

## 5 Experiments results and analysis

The proposed method is validated and applied using experiments from two aspects. First, a series of benchmark function experiments are conducted to validate the performance of GACO, and second, bGACO-SVM is applied to some classification prediction problems on feature selection, which effectively illustrates that bGACO-SVM has strong classification prediction capability.

### 5.1 Benchmark function validation

In this subsection, IEEE CEC2017 is used as the basis for benchmark function experiments, and the core advantages of GACO with strong convergence performance and less susceptibility to local optima are fully illustrated by comparing experiments not only between GACO and nine basic algorithms, but also between GACO and nine advanced variant algorithms.

#### 5.1.1 Experiment setup

In the benchmark function experiments, 30 benchmark functions in IEEE CEC2017 are used as the basis of the experiments, which are illustrated in Table 5. In the process of the experiments, the basic algorithm comparative experiments and advanced variant algorithm comparative experiments were conducted. In the basic algorithm comparative experiments, the algorithms involved in the simulation are ACOR (Socha and Dorigo, 2008), GWO (Mirjalili et al., 2014), HHO (Heidari et al., 2019), MFO (Mirjalili, 2015), PSO (Kennedy and Eberhart, 1995), WOA (Mirjalili and Lewis, 2016), JAYA (Rao, 2016), FA (Yang, 2009), and SFS (Salimi, 2015), whose superior performance has been well demonstrated in some previous original studies, so much so that the comparison with their comparative results are also convincing. It is also necessary to compare with some advanced variant algorithms since GACO actually belongs to one variant algorithm, where MOFOA (Chen et al., 2020), CDLOBA (Yong et al., 2018), HGWO (Zhu et al., 2015), OBSCA (Abd Elaziz et al., 2017), SMFO (Chen C. et al., 2021), CGSCA (Chen C. et al., 2021), RDWOA (Chen et al., 2019), m_SCA (Qu et al., 2018), and BMWOA (Heidari et al., 2020) participate in the comparative study. These variant algorithms involved in the comparison not only have a performance due to but also have been successfully applied to several fields.

**TABLE 5:** The detailed 30 benchmark functions of CEC2017.

Item	The function class	The function name	The optimal fitness
F1	Unimodal functions	Shifted and rotated bent cigar function	100
F2		Shifted and rotated sum of different power function	200
F3		Shifted and rotated zakharov function	300
F4	Multimodal functions	Shifted and rotated rosenbrocks function	400
F5		Shifted and rotated rastrigins function	500
F6		Shifted and rotated expanded scaffers F6 function	600
F7		Shifted and rotated lunacek Bi_Rastrigin function	700
F8		Shifted and rotated Non-continuous rastrigins function	800
F9		Shifted and rotated levy function	900
F10		Shifted and rotated schwefels function	1,000
F11	Hybrid functions	Hybrid function 1 (*N* = 3)	1,100
F12		Hybrid function 2 (*N* = 3)	1,200
F13		Hybrid function 3 (*N* = 3)	1,300
F14		Hybrid function 4 (*N* = 4)	1,400
F15		Hybrid function 5 (*N* = 4)	1,500
F16		Hybrid function 6 (*N* = 4)	1,600
F17		Hybrid function 6 (*N* = 5)	1,700
F18		Hybrid function 6 (*N* = 5)	1,800
F19		Hybrid function 6 (*N* = 5)	1,900
F20		Hybrid function 6 (*N* = 6)	2,000
F21	Composition functions	Composition function 1 (*N* = 3)	2,100
F22		Composition function 2 (*N* = 3)	2,200
F23		Composition function 3 (*N* = 4)	2,300
F24		Composition function 4 (*N* = 4)	2,400
F25		Composition function 5 (*N* = 5)	2,500
F26		Composition function 6 (*N* = 5)	2,600
F27		Composition function 7 (*N* = 6)	2,700
F28		Composition function 8 (*N* = 6)	2,800
F29		Composition function 9 (*N* = 3)	2,900
F30		Composition function 10 (*N* = 3)	3,000

Furthermore, to ensure fairness of the experimental process and the accuracy of the experimental results, all the algorithms participating in the comparison are carried out under the same conditions, where the population size is set to 30 and the maximum number of evaluations is uniformly set to 300,000. In addition, the values of the key parameters of all the algorithms involved in the comparison are kept consistent with the values of the parameters in the original literature. Besides, all algorithms are independently tested 30 times to reduce the effect of random conditions. Mean, standard deviation, the Wilcoxon signed-rank test and the Friedman test are used for detailed statistics and analysis of all experimental results obtained on the benchmark functions.

#### 5.1.2 Comparison with basic algorithms

In this subsection, GACO and nine basic algorithms are compared in experiments at IEEE CEC2017, and the algorithms involved in the comparison are ACOR (Socha and Dorigo, 2008), GWO (Mirjalili et al., 2014), HHO (Heidari et al., 2019), MFO (Mirjalili, 2015), PSO (Kennedy and Eberhart, 1995), WOA (Mirjalili and Lewis, 2016), JAYA (Rao, 2016), FA (Yang, 2009), and SFS (Salimi, 2015). Table 6 gives their average values and standard deviations obtained during the experiment, where “AVG” denotes the average value and “STD” denotes the standard deviation. The best results are bolded in each column. By observing the average and standard deviation, it can be found that GACO obtained the minimum average value on 20 functions, PSO on 6 functions and GWO on 4 functions. Based on the observation of the average values, it is evident that GACO performs the best on two-thirds of the functions, which effectively shows that GACO has a strong optimization capability to obtain high-quality solutions. Similarly, GACO also performs well in terms of standard deviation, indicating that it has good stability.

**TABLE 6:** The average values and the standard deviations obtained by GACO and basic algorithms.

	F1		F2		F3	
	AVG	STD	AVG	STD	AVG	STD
GACO	**8.2256E + 06**	**7.7175E + 06**	1.0590E + 85	5.0960E + 85	3.0497E + 05	5.4569E + 04
ACOR	3.6100E + 10	1.7715E + 10	3.0027E + 138	1.6446E + 139	6.0684E + 05	9.0501E + 04
GWO	5.0960E + 10	1.0219E + 10	3.6382E + 132	1.9923E + 133	2.3043E + 05	2.0478E + 04
HHO	4.3145E + 08	4.1722E + 07	4.0250E + 96	1.2975E + 97	1.9619E + 05	1.9851E + 04
MFO	1.4511E + 11	5.9631E + 10	1.0574E + 164	**6.5535E + 04**	6.3817E + 05	1.8557E + 05
PSO	1.6059E + 09	9.8073E + 07	**8.2461E + 74**	2.6019E + 75	**8.4109E + 04**	1.5101E + 04
WOA	1.2008E + 09	4.6558E + 08	2.2068E + 152	1.0509E + 153	7.8453E + 05	1.5669E + 05
JAYA	1.0349E + 11	1.1484E + 10	4.1537E + 141	2.2577E + 142	3.9396E + 05	4.0375E + 04
FA	1.2877E + 11	7.0207E + 09	7.4327E + 151	2.2871E + 152	4.3255E + 05	3.2019E + 04
SFS	4.7679E + 10	9.3071E + 09	1.5726E + 134	8.5640E + 134	2.4843E + 05	**1.4207E + 04**

	**F4**		**F5**		**F6**	
	**AVG**	**STD**	**AVG**	**STD**	**AVG**	**STD**

GACO	**7.1068E + 02**	**5.5357E + 01**	**1.0100E + 03**	2.8355E + 02	**6.0957E + 02**	3.2392E + 00
ACOR	3.2978E + 03	1.7649E + 03	1.7046E + 03	1.4535E + 02	6.2972E + 02	4.8767E + 00
GWO	4.9131E + 03	1.3196E + 03	1.1389E + 03	6.5861E + 01	6.3857E + 02	5.6532E + 00
HHO	1.0370E + 03	9.1619E + 01	1.4964E + 03	5.5559E + 01	6.8146E + 02	4.4131E + 00
MFO	2.9679E + 04	1.5588E + 04	1.8138E + 03	1.6297E + 02	6.7074E + 02	5.3080E + 00
PSO	7.7564E + 02	1.0928E + 02	1.6982E + 03	7.1390E + 01	6.9035E + 02	6.6557E + 00
WOA	1.5272E + 03	1.8335E + 02	1.4911E + 03	8.8708E + 01	6.8498E + 02	8.6502E + 00
JAYA	1.7696E + 04	2.7371E + 03	1.7631E + 03	5.6782E + 01	6.7036E + 02	6.0310E + 00
FA	1.7797E + 04	1.8868E + 03	1.7553E + 03	**3.0198E + 01**	6.7541E + 02	**2.5695E + 00**
SFS	6.0276E + 03	1.3589E + 03	1.4953E + 03	4.5356E + 01	6.6920E + 02	5.6041E + 00

	**F7**		**F8**		**F9**	
	**AVG**	**STD**	**AVG**	**STD**	**AVG**	**STD**

GACO	**1.6294E + 03**	2.9561E + 02	**1.3525E + 03**	3.0638E + 02	**1.5681E + 04**	5.0118E + 03
ACOR	3.3360E + 03	4.6313E + 02	1.8264E + 03	2.8730E + 02	6.8794E + 04	1.5724E + 04
GWO	1.9769E + 03	1.5215E + 02	1.4535E + 03	5.3471E + 01	2.6998E + 04	1.1298E + 04
HHO	3.6528E + 03	1.5064E + 02	1.9509E + 03	5.5012E + 01	4.6558E + 04	4.7553E + 03
MFO	5.2019E + 03	1.1267E + 03	2.2185E + 03	1.5174E + 02	4.8050E + 04	4.6064E + 03
PSO	1.8430E + 03	**6.3657E + 01**	2.0805E + 03	8.6441E + 01	6.7596E + 04	6.9463E + 03
WOA	3.3797E + 03	1.7804E + 02	1.9691E + 03	9.5630E + 01	4.0896E + 04	8.6796E + 03
JAYA	3.0867E + 03	1.6241E + 02	2.1500E + 03	6.4354E + 01	4.9478E + 04	5.5002E + 03
FA	4.9452E + 03	2.0113E + 02	2.0569E + 03	**3.2362E + 01**	4.9924E + 04	**2.4190E + 03**
SFS	2.7896E + 03	2.6812E + 02	1.9160E + 03	8.0118E + 01	5.4216E + 04	7.1330E + 03

	**F10**		**F11**		**F12**	
	**AVG**	**STD**	**AVG**	**STD**	**AVG**	**STD**

GACO	3.0986E + 04	3.2558E + 03	3.9846E + 03	1.3114E + 03	**9.9691E + 07**	**8.6298E + 07**
ACOR	3.1319E + 04	9.4427E + 02	3.6931E + 04	3.1612E + 04	2.3767E + 09	3.5117E + 09
GWO	**1.4934E + 04**	1.3572E + 03	5.3227E + 04	1.4592E + 04	9.7268E + 09	3.8915E + 09
HHO	2.0134E + 04	1.4566E + 03	4.7016E + 03	8.0608E + 02	5.1398E + 08	1.6789E + 08
MFO	1.7023E + 04	1.6742E + 03	1.3476E + 05	8.1816E + 04	4.0575E + 10	2.2988E + 10

	**F10**		**F11**		**F12**	
	**AVG**	**STD**	**AVG**	**STD**	**AVG**	**STD**

PSO	2.4828E + 04	1.2067E + 03	**3.5492E + 03**	**2.3929E + 02**	1.0644E + 09	1.9690E + 08
WOA	2.3319E + 04	2.5357E + 03	5.9014E + 04	3.5771E + 04	1.2011E + 09	4.0590E + 08
JAYA	3.1447E + 04	5.8932E + 02	6.2055E + 04	9.2973E + 03	2.7457E + 10	4.7948E + 09
FA	3.1171E + 04	**4.4500E + 02**	1.2679E + 05	1.7575E + 04	4.7380E + 10	3.3462E + 09
SFS	2.3193E + 04	3.0843E + 03	4.7273E + 04	9.6927E + 03	1.0455E + 10	5.7766E + 09

	**F13**		**F14**		**F15**	
	**AVG**	**STD**	**AVG**	**STD**	**AVG**	**STD**

GACO	**9.4264E + 04**	**1.1070E + 05**	**7.7696E + 05**	8.1489E + 05	**1.7601E + 04**	**1.1305E + 04**
ACOR	8.6415E + 07	3.2235E + 08	1.2860E + 06	2.8603E + 06	9.9486E + 07	5.4309E + 08
GWO	1.0131E + 09	7.3267E + 08	5.7396E + 06	3.2209E + 06	1.9895E + 08	3.0217E + 08
HHO	7.2212E + 06	1.2380E + 06	2.0307E + 06	5.5807E + 05	2.3151E + 06	1.3174E + 06
MFO	6.7366E + 09	5.2358E + 09	8.8703E + 06	1.0445E + 07	1.6566E + 09	2.3418E + 09
PSO	9.4036E + 07	9.1179E + 06	1.4371E + 06	**4.8151E + 05**	3.2478E + 07	4.2943E + 06
WOA	1.0125E + 06	6.5198E + 05	4.0841E + 06	2.1279E + 06	6.5230E + 05	1.5870E + 06
JAYA	3.3460E + 09	5.8203E + 08	2.0404E + 07	6.1547E + 06	1.2102E + 09	2.2209E + 08
FA	9.7405E + 09	8.4986E + 08	4.0528E + 07	9.8837E + 06	3.8676E + 09	5.7707E + 08
SFS	5.3077E + 08	7.4002E + 08	4.3394E + 06	2.0366E + 06	7.6357E + 06	5.2135E + 06

	**F16**		**F17**		**F18**	
	**AVG**	**STD**	**AVG**	**STD**	**AVG**	**STD**
	
GACO	**5.7358E + 03**	2.3390E + 03	6.4921E + 03	1.7745E + 03	4.4177E + 06	2.3376E + 06
ACOR	6.7722E + 03	2.4763E + 03	5.7058E + 03	1.2735E + 03	3.5441E + 06	2.1597E + 06
GWO	6.0808E + 03	5.9306E + 02	**5.3245E + 03**	9.8942E + 02	4.0363E + 06	2.0454E + 06
HHO	7.5667E + 03	8.4357E + 02	6.2019E + 03	5.9569E + 02	3.2626E + 06	1.2479E + 06
MFO	8.2079E + 03	1.0619E + 03	1.1898E + 04	8.5543E + 03	1.8763E + 07	2.3755E + 07
PSO	8.0057E + 03	6.9435E + 02	6.0817E + 03	**5.1887E + 02**	**2.8781E + 06**	**1.1508E + 06**
WOA	1.2021E + 04	1.5446E + 03	7.5235E + 03	8.9234E + 02	3.7969E + 06	1.9433E + 06
JAYA	1.1935E + 04	3.9134E + 02	1.0884E + 04	6.3055E + 02	3.1558E + 07	1.0144E + 07
FA	1.1908E + 04	**3.3078E + 02**	1.2672E + 04	1.2568E + 03	6.7360E + 07	1.5507E + 07
SFS	8.2635E + 03	9.8760E + 02	6.1484E + 03	8.7472E + 02	5.7382E + 06	3.5855E + 06

	**F19**		**F20**		**F21**	
	**AVG**	**STD**	**AVG**	**STD**	**AVG**	**STD**

GACO	**9.0580E + 04**	**1.4671E + 05**	6.7341E + 03	1.2898E + 03	**2.8239E + 03**	2.6964E + 02
ACOR	2.3778E + 07	1.2803E + 08	6.8177E + 03	9.5926E + 02	3.4864E + 03	2.3384E + 02
GWO	2.9512E + 08	5.5053E + 08	**4.7312E + 03**	7.1369E + 02	2.9656E + 03	8.0566E + 01
HHO	7.9411E + 06	3.5798E + 06	5.8085E + 03	4.1462E + 02	4.0340E + 03	2.0346E + 02
MFO	8.6606E + 08	1.0386E + 09	5.8370E + 03	6.9192E + 02	3.7331E + 03	1.6980E + 02
PSO	5.0654E + 07	1.0964E + 07	5.9648E + 03	4.7727E + 02	3.8360E + 03	1.3323E + 02
WOA	2.6514E + 07	1.6930E + 07	6.3206E + 03	4.8385E + 02	3.9557E + 03	2.3823E + 02
JAYA	1.2052E + 09	2.3756E + 08	7.2429E + 03	2.3323E + 02	3.5848E + 03	5.3667E + 01
FA	3.9109E + 09	5.0919E + 08	7.1081E + 03	**2.2258E + 02**	3.5997E + 03	**2.7543E + 01**
SFS	3.5426E + 07	4.1572E + 07	5.6331E + 03	3.9694E + 02	3.5285E + 03	1.1670E + 02

	**F22**		**F23**		**F24**	
	**AVG**	**STD**	**AVG**	**STD**	**AVG**	**STD**

GACO	3.2237E + 04	4.7406E + 03	**3.2363E + 03**	1.6394E + 02	**3.8665E + 03**	2.8500E + 02
ACOR	3.2137E + 04	3.5784E + 03	3.4544E + 03	1.2518E + 02	3.9442E + 03	1.5828E + 02
GWO	**1.9020E + 04**	3.3369E + 03	3.5578E + 03	8.2209E + 01	4.1983E + 03	9.2443E + 01
HHO	2.3925E + 04	1.5476E + 03	5.2012E + 03	2.8788E + 02	6.3045E + 03	4.0125E + 02
MFO	2.0418E + 04	1.6253E + 03	3.8749E + 03	1.4992E + 02	4.5054E + 03	1.7933E + 02
PSO	2.8077E + 04	1.2457E + 03	4.9337E + 03	2.7125E + 02	5.8868E + 03	4.9029E + 02
WOA	2.5579E + 04	2.4395E + 03	4.7940E + 03	3.4278E + 02	6.0490E + 03	3.6440E + 02
JAYA	3.3901E + 04	5.7469E + 02	4.6835E + 03	1.4235E + 02	6.1066E + 03	2.1815E + 02
FA	3.3445E + 04	**5.2014E + 02**	4.1180E + 03	**3.7619E + 01**	4.8621E + 03	**5.9943E + 01**
SFS	2.5187E + 04	2.8835E + 03	4.4516E + 03	1.9623E + 02	5.8170E + 03	2.5337E + 02

	**F25**		**F26**		**F27**	
	**AVG**	**STD**	**AVG**	**STD**	**AVG**	**STD**

GACO	**3.3714E + 03**	5.8312E + 01	**1.0976E + 04**	2.9287E + 03	3.5413E + 03	6.2898E + 01
ACOR	6.1377E + 03	1.6769E + 03	1.3491E + 04	1.3763E + 03	3.6055E + 03	9.8467E + 01
GWO	6.3944E + 03	1.1488E + 03	1.5364E + 04	1.3283E + 03	4.0608E + 03	1.2048E + 02
HHO	3.6669E + 03	1.0497E + 02	2.3951E + 04	5.5858E + 03	4.4867E + 03	4.3769E + 02
MFO	1.3739E + 04	6.5349E + 03	1.9147E + 04	1.6406E + 03	4.1285E + 03	2.6704E + 02
PSO	3.4591E + 03	**5.1934E + 01**	1.6941E + 04	8.3486E + 03	**3.2845E + 03**	**4.2689E + 01**
WOA	4.0227E + 03	1.1089E + 02	3.1718E + 04	3.0965E + 03	5.3490E + 03	1.0257E + 03
JAYA	1.1044E + 04	1.1947E + 03	3.1820E + 04	1.8720E + 03	6.4061E + 03	4.0874E + 02
FA	1.8005E + 04	1.3440E + 03	2.1482E + 04	**5.1965E + 02**	5.2925E + 03	1.6817E + 02
SFS	6.3670E + 03	6.0614E + 02	2.7142E + 04	2.9023E + 03	5.2094E + 03	3.8056E + 02

	**F28**		**F29**		**F30**	
	**AVG**	**STD**	**AVG**	**STD**	**AVG**	**STD**

GACO	3.5733E + 03	5.2075E + 02	**6.2878E + 03**	5.4587E + 02	**1.0256E + 06**	**1.7309E + 06**
ACOR	1.5202E + 04	1.8765E + 03	6.9565E + 03	7.5323E + 02	1.9251E + 08	7.2647E + 08
GWO	8.5347E + 03	1.6676E + 03	8.1485E + 03	8.8332E + 02	9.4571E + 08	9.9770E + 08
HHO	3.6801E + 03	6.3656E + 01	9.6652E + 03	6.7693E + 02	5.1889E + 07	1.8154E + 07
MFO	2.1062E + 04	2.2769E + 03	1.1910E + 04	4.4811E + 03	2.7856E + 09	2.4137E + 09
PSO	**3.4322E + 03**	**5.4773E + 01**	9.6976E + 03	**4.5820E + 02**	1.4845E + 08	3.5743E + 07
WOA	4.3863E + 03	2.3776E + 02	1.4509E + 04	1.8289E + 03	4.2799E + 08	1.8114E + 08
JAYA	1.9079E + 04	1.7054E + 03	1.3307E + 04	6.8642E + 02	2.2355E + 09	4.1862E + 08
FA	1.6331E + 04	1.3891E + 03	1.8595E + 04	1.5208E + 03	6.0185E + 09	6.2062E + 08
SFS	9.0535E + 03	1.1890E + 03	1.0488E + 04	8.3307E + 02	7.9833E + 08	1.2287E + 09

Further, Table 7 gives the performance ranking of all algorithms on each benchmark function, as well as the overall ranking on the 30 benchmark functions. The observation shows that GACO not only performs as No. 1 on every function category in IEEE CEC2017, but it also ranks No. 1 overall on 30 functions, which provides sufficient proof of GACO’s performance. In addition, for a more reliable analysis of the experimental results, the results of the Wilcoxon signed-rank test are presented in Table 7, where “+” indicates that GACO outperforms the comparison algorithm, “-” indicates that GACO outperforms the comparison algorithm, and “=” indicates that the performance of GACO and the comparison algorithm are comparable. Based on the observation of the analysis results, GACO outperforms the other algorithms on 20 out of 30 functions, which provides sufficient evidence to prove the performance of GACO.

**TABLE 7:** The performance ranking of all algorithms and the results of the Wilcoxon signed-rank test.

F	GACO	ACOR	GWO	HHO	MFO	PSO	WOA	JAYA	FA	SFS
F1	1	5	7	2	10	4	3	8	9	6
F2	2	6	4	3	10	1	9	7	8	5
F3	5	8	3	2	9	1	10	6	7	4
F4	1	5	6	3	10	2	4	8	9	7
F5	1	7	2	5	10	6	3	9	8	4
F6	1	2	3	8	6	10	9	5	7	4
F7	1	6	3	8	10	2	7	5	9	4
F8	1	3	2	5	10	8	6	9	7	4
F9	1	10	2	4	5	9	3	6	7	8
F10	7	9	1	3	2	6	5	10	8	4
F11	2	4	6	3	10	1	7	8	9	5
F12	1	5	6	2	9	3	4	8	10	7
F13	1	4	7	3	9	5	2	8	10	6
F14	1	2	7	4	8	3	5	9	10	6
F15	1	6	7	3	9	5	2	8	10	4
F16	1	3	2	4	6	5	10	9	8	7
F17	6	2	1	5	9	3	7	8	10	4
F18	6	3	5	2	8	1	4	9	10	7
F19	1	3	7	2	8	6	4	9	10	5
F20	7	8	1	3	4	5	6	10	9	2
F21	1	3	2	10	7	8	9	5	6	4
F22	8	7	1	3	2	6	5	10	9	4
F23	1	2	3	10	4	9	8	7	5	6
F24	1	2	3	10	4	7	8	9	5	6
F25	1	5	7	3	9	2	4	8	10	6
F26	1	2	3	7	5	4	9	10	6	8
F27	2	3	4	6	5	1	9	10	8	7
F28	2	7	5	3	10	1	4	9	8	6
F29	1	2	3	4	7	5	9	8	10	6
F30	1	4	7	2	9	3	5	8	10	6
± / =	∼	22/0/8	21/5/4	24/5/1	27/3/0	20/8/2	26/3/1	28/0/2	28/0/2	24/4/2
Mean	2.23	4.60	4.00	4.40	7.47	4.40	6.00	8.10	8.40	5.40
Rank	1	5	2	3	8	3	7	9	10	6

**FIGURE 3:**
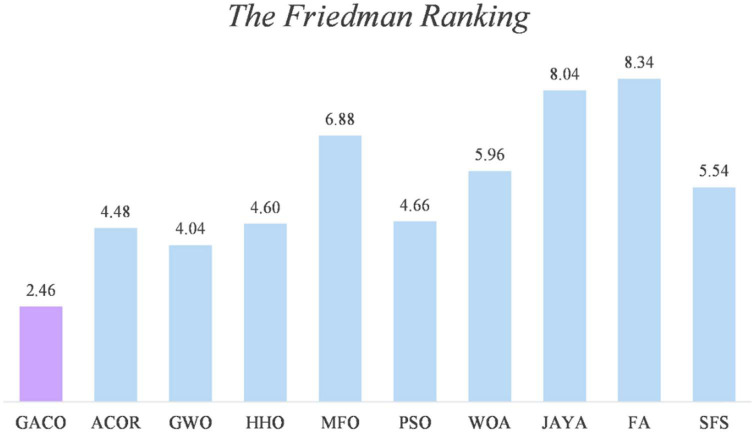
The results of the Friedman test analysis.

**FIGURE 4:**
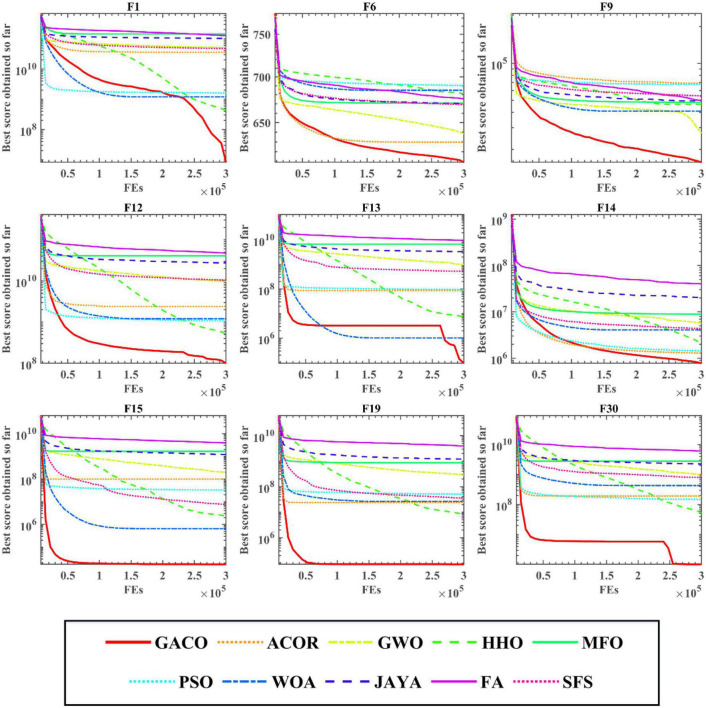
The convergence curves of GACO and basic algorithms on some functions.

After the Wilcoxon signed-rank test analysis, Figure 3 gives the results of the Friedman test analysis, where GACO is No. 1 with 2.46 and GWO is No. 2 with 4.04. Therefore, the analysis shows that GACO has a greater advantage over GWO, which is ranked No. 2, and this also shows that GACO has an advantage over other algorithms. Finally, to illustrate the convergence performance of GACO, the convergence curves on some functions are given in Figure 4, where F6, F9, F14, F15, and F19 demonstrate that the convergence performance of GACO is better than other basic algorithms, and F1, F12, F13, and F20 reflect the strong ability of avoiding falling into local optimum that GACO has.

Therefore, based on the above experimental analysis, the convergence performance of GACO, as well as the stronger ability to avoid local optima, are well demonstrated in the comparison experiments of the basic algorithms.

#### 5.1.3 Comparison with state-of-art variants

This subsection compares GACO with nine advanced variants since GACO is one variant by introducing the GS strategy into ACOR. Their average values and standard deviations obtained for each function are given in Table 8, where the best results are bolded in each column, GACO obtains the best average value on 23 functions, CDLOBA obtains the best average value on 5 functions, and m_SCA has a better performance on 2 two functions. Based on the observation of the mean performance, it is clear that GACO has a very obvious advantage over other algorithms. In addition, its performance on STD is also very good, which also reflects that GACO has some stability.

**TABLE 8:** The average values and standard deviations of GACO and nine advanced variants.

	F1		F2		F3	
	AVG	STD	AVG	STD	AVG	STD
GACO	**5.6891E + 06**	**4.3937E + 06**	**7.4428E + 89**	4.0760E + 90	3.1735E + 05	3.8486E + 04
MOFOA	2.6711E + 11	2.6295E + 09	1.1255E + 170	**6.5535E + 04**	3.3772E + 05	**4.2760E + 03**
CDLOBA	1.2623E + 07	4.8252E + 07	1.2300E + 108	6.7369E + 108	3.6842E + 05	1.1282E + 05
HGWO	1.0067E + 11	7.5732E + 09	3.6954E + 143	2.0052E + 144	3.3992E + 05	1.4764E + 04
OBSCA	1.9600E + 11	1.2115E + 10	3.7517E + 163	6.5535E + 04	3.5362E + 05	2.4996E + 04
SMFO	2.2737E + 11	2.1089E + 10	9.8487E + 172	6.5535E + 04	3.5021E + 05	1.5963E + 04
CGSCA	1.7607E + 11	7.6925E + 09	1.0114E + 153	6.5535E + 04	3.0041E + 05	1.4008E + 04
RDWOA	2.7916E + 09	2.7871E + 09	7.2820E + 123	3.2861E + 124	2.4424E + 05	2.3924E + 04
m_SCA	1.1729E + 11	1.4978E + 10	4.8033E + 144	2.6309E + 145	**2.3064E + 05**	2.3654E + 04
BMWOA	2.4823E + 11	1.3003E + 10	1.4963E + 171	6.5535E + 04	3.9269E + 05	8.5130E + 04

	**F4**		**F5**		**F6**	
	**AVG**	**STD**	**AVG**	**STD**	**AVG**	**STD**

GACO	**7.1727E + 02**	**5.4114E + 01**	**9.0318E + 02**	2.0575E + 02	**6.0762E + 02**	2.7936E + 00
MOFOA	9.9043E + 04	2.9035E + 03	2.1535E + 03	**1.8140E + 01**	7.1991E + 02	**9.8085E-01**
CDLOBA	9.7324E + 02	1.3149E + 02	2.0058E + 03	1.5369E + 02	6.8393E + 02	6.2058E + 00
HGWO	1.1036E + 04	1.0112E + 03	1.7152E + 03	3.0153E + 01	6.7553E + 02	2.4073E + 00
OBSCA	4.1876E + 04	5.3045E + 03	1.9819E + 03	5.1167E + 01	6.9791E + 02	4.3750E + 00
SMFO	7.1560E + 04	1.1179E + 04	2.0341E + 03	5.1684E + 01	7.0666E + 02	6.2014E + 00
CGSCA	3.6263E + 04	5.3517E + 03	1.9653E + 03	5.3088E + 01	6.9753E + 02	4.5596E + 00
RDWOA	1.3230E + 03	1.9472E + 02	1.5463E + 03	1.0643E + 02	6.6650E + 02	6.8233E + 00
m_SCA	1.3195E + 04	3.5938E + 03	1.4408E + 03	8.6192E + 01	6.6553E + 02	3.9864E + 00
BMWOA	8.3366E + 04	8.2941E + 03	2.1573E + 03	3.7247E + 01	7.1575E + 02	2.0205E + 00

	**F7**		**F8**		**F9**	
	**AVG**	**STD**	**AVG**	**STD**	**AVG**	**STD**

GACO	**1.6553E + 03**	2.9269E + 02	**1.3096E + 03**	2.9279E + 02	**1.6619E + 04**	5.2477E + 03
MOFOA	4.0402E + 03	**2.8747E + 01**	2.6259E + 03	**1.7593E + 01**	8.5000E + 04	**2.4664E + 03**
CDLOBA	1.0446E + 04	6.6761E + 02	2.5220E + 03	1.2641E + 02	4.7612E + 04	5.3404E + 03
HGWO	2.7900E + 03	8.5229E + 01	2.0427E + 03	3.0169E + 01	5.4724E + 04	4.3961E + 03
OBSCA	3.7478E + 03	1.1665E + 02	2.3505E + 03	7.0773E + 01	7.7112E + 04	5.5740E + 03
SMFO	3.9405E + 03	8.1444E + 01	2.5141E + 03	7.8276E + 01	7.2056E + 04	4.2652E + 03
CGSCA	3.4932E + 03	1.0714E + 02	2.3359E + 03	6.8760E + 01	7.4300E + 04	2.8601E + 03
RDWOA	3.0339E + 03	2.0416E + 02	2.0010E + 03	1.6488E + 02	3.0604E + 04	6.8706E + 03
m_SCA	2.8941E + 03	1.8522E + 02	1.7863E + 03	7.0689E + 01	3.9727E + 04	7.3789E + 03
BMWOA	4.0752E + 03	8.5327E + 01	2.6241E + 03	4.6038E + 01	8.7045E + 04	4.1294E + 03

	**F10**		**F11**		**F12**	
	**AVG**	**STD**	**AVG**	**STD**	**AVG**	**STD**

GACO	3.1032E + 04	3.5120E + 03	**4.6920E + 03**	**2.2564E + 03**	9.5070E + 07	8.5116E + 07
MOFOA	3.1963E + 04	**4.9890E + 02**	2.0592E + 05	1.5329E + 04	2.1472E + 11	5.2516E + 09
CDLOBA	**1.6908E + 04**	1.4985E + 03	6.9745E + 03	3.6119E + 03	**4.9396E + 07**	**4.0021E + 07**
HGWO	2.6578E + 04	7.9503E + 02	1.6128E + 05	1.5017E + 04	2.8297E + 10	3.5749E + 09
OBSCA	2.9336E + 04	1.1102E + 03	1.1464E + 05	1.3140E + 04	8.5411E + 10	8.6090E + 09

	**F10**		**F11**		**F12**	
	**AVG**	**STD**	**AVG**	**STD**	**AVG**	**STD**

SMFO	3.1746E + 04	1.0942E + 03	2.5603E + 05	8.5753E + 04	1.3429E + 11	2.7382E + 10
CGSCA	3.1376E + 04	5.6440E + 02	1.0540E + 05	1.5132E + 04	7.1672E + 10	8.4520E + 09
RDWOA	2.0674E + 04	2.3922E + 03	1.6766E + 04	5.1345E + 03	5.9383E + 08	5.5529E + 08
m_SCA	1.9045E + 04	1.9004E + 03	4.7959E + 04	1.0684E + 04	2.8088E + 10	8.8816E + 09
BMWOA	3.3092E + 04	6.1772E + 02	3.4479E + 05	8.4359E + 04	1.6690E + 11	1.4943E + 10

	**F13**		**F14**		**F15**	
	**AVG**	**STD**	**AVG**	**STD**	**AVG**	**STD**

GACO	**6.6817E + 04**	7.9597E + 04	9.4596E + 05	5.7572E + 05	**1.7428E + 04**	**1.2184E + 04**
MOFOA	5.2952E + 10	1.0296E + 09	1.4727E + 08	5.2422E + 07	3.0293E + 10	2.5254E + 09
CDLOBA	1.0030E + 05	**3.2683E + 04**	**1.5607E + 05**	**8.3605E + 04**	1.0944E + 05	5.4513E + 04
HGWO	5.5367E + 09	7.5157E + 08	1.7214E + 07	5.4166E + 06	1.5541E + 09	4.7492E + 08
OBSCA	1.5458E + 10	2.2157E + 09	3.1149E + 07	1.1222E + 07	4.8525E + 09	1.3541E + 09
SMFO	2.8976E + 10	6.3323E + 09	4.8643E + 07	2.8715E + 07	1.2242E + 10	4.7235E + 09
CGSCA	1.1377E + 10	1.9100E + 09	1.9230E + 07	4.7376E + 06	3.5175E + 09	8.0500E + 08
RDWOA	4.5217E + 06	8.8377E + 06	1.2513E + 06	5.4704E + 05	5.0743E + 04	1.1522E + 05
m_SCA	3.6031E + 09	1.6165E + 09	5.6029E + 06	3.4815E + 06	7.8553E + 08	9.0421E + 08
BMWOA	3.5298E + 10	5.4313E + 09	6.0155E + 07	2.6975E + 07	1.6107E + 10	3.6335E + 09

	**F16**		**F17**		**F18**	
	**AVG**	**STD**	**AVG**	**STD**	**AVG**	**STD**

GACO	**5.9147E + 03**	2.5403E + 03	**5.8620E + 03**	1.6837E + 03	4.8110E + 06	1.9265E + 06
MOFOA	2.3638E + 04	1.2695E + 03	1.6826E + 07	4.5661E + 06	3.1738E + 08	6.3057E + 07
CDLOBA	8.0546E + 03	1.1168E + 03	6.8603E + 03	**6.1523E + 02**	**3.2821E + 05**	**1.7020E + 05**
HGWO	1.0611E + 04	**5.2572E + 02**	9.8283E + 03	1.4170E + 03	2.1173E + 07	6.3602E + 06
OBSCA	1.4051E + 04	6.7942E + 02	2.5773E + 04	1.0563E + 04	5.1867E + 07	2.5038E + 07
SMFO	1.9104E + 04	3.1393E + 03	1.9592E + 06	3.2793E + 06	9.2659E + 07	8.8024E + 07
CGSCA	1.3661E + 04	6.9149E + 02	1.9455E + 04	1.0214E + 04	3.3627E + 07	1.4577E + 07
RDWOA	8.5543E + 03	8.7235E + 02	6.3095E + 03	8.2113E + 02	2.3249E + 06	1.0906E + 06
m_SCA	7.7833E + 03	9.3556E + 02	5.9623E + 03	7.7630E + 02	7.3133E + 06	3.9826E + 06
BMWOA	1.9585E + 04	1.7580E + 03	2.4042E + 06	1.9447E + 06	1.3773E + 08	5.7575E + 07

	**F19**		**F20**		**F21**	
	**AVG**	**STD**	**AVG**	**STD**	**AVG**	**STD**

GACO	**7.1769E + 04**	**1.0313E + 05**	6.1385E + 03	1.6546E + 03	**2.7472E + 03**	2.1729E + 02
MOFOA	3.0201E + 10	5.4381E + 08	8.1863E + 03	**1.7316E + 02**	5.3523E + 03	1.7323E + 02
CDLOBA	5.4223E + 05	1.1564E + 05	6.1509E + 03	6.6181E + 02	4.1918E + 03	2.2466E + 02
HGWO	1.4594E + 09	3.4800E + 08	6.5100E + 03	3.7159E + 02	3.5718E + 03	**4.1709E + 01**
OBSCA	4.0578E + 09	1.2242E + 09	6.8729E + 03	3.1138E + 02	4.0106E + 03	8.8106E + 01
SMFO	9.2857E + 09	3.7734E + 09	7.1751E + 03	3.4021E + 02	4.5424E + 03	1.8386E + 02
CGSCA	3.2830E + 09	7.6698E + 08	7.2223E + 03	2.6327E + 02	4.0227E + 03	8.6903E + 01
RDWOA	1.9957E + 05	2.0600E + 05	5.7283E + 03	5.2369E + 02	3.6490E + 03	2.3422E + 02
m_SCA	8.4100E + 08	6.9085E + 08	**5.2470E + 03**	6.2811E + 02	3.3516E + 03	1.0000E + 02
BMWOA	1.3745E + 10	2.7376E + 09	8.1658E + 03	3.1767E + 02	4.5849E + 03	1.1633E + 02

	**F22**		**F23**		**F24**	
	**AVG**	**STD**	**AVG**	**STD**	**AVG**	**STD**

GACO	3.3499E + 04	6.7654E + 02	**3.2564E + 03**	2.3647E + 02	**3.7234E + 03**	2.1216E + 02
MOFOA	3.6376E + 04	**4.1795E + 02**	6.7876E + 03	2.8532E + 02	1.1421E + 04	1.1510E + 03
CDLOBA	**1.9533E + 04**	1.1609E + 03	5.1652E + 03	3.1021E + 02	7.0944E + 03	5.0849E + 02
HGWO	2.0952E + 04	6.3594E + 03	4.2797E + 03	**4.7083E + 01**	5.1699E + 03	**1.1019E + 02**
OBSCA	2.9696E + 04	1.6673E + 03	5.0120E + 03	1.0476E + 02	6.7822E + 03	2.3221E + 02
SMFO	3.3642E + 04	8.8573E + 02	6.3810E + 03	4.2232E + 02	1.0113E + 04	1.0687E + 03
CGSCA	3.3714E + 04	1.4690E + 03	4.8181E + 03	1.1706E + 02	6.2838E + 03	2.0506E + 02
RDWOA	2.3229E + 04	2.3558E + 03	4.0729E + 03	2.0916E + 02	4.9384E + 03	3.2467E + 02
m_SCA	2.1217E + 04	1.5259E + 03	3.9738E + 03	1.0417E + 02	4.9441E + 03	1.9369E + 02
BMWOA	3.6109E + 04	9.8353E + 02	5.5581E + 03	2.1316E + 02	8.4928E + 03	7.5663E + 02

	**F25**		**F26**		**F27**	
	**AVG**	**STD**	**AVG**	**STD**	**AVG**	**STD**

GACO	**3.3748E + 03**	**6.7374E + 01**	**1.1059E + 04**	3.0245E + 03	**3.5337E + 03**	**7.3582E + 01**
MOFOA	2.6610E + 04	9.4632E + 02	5.6693E + 04	9.5579E + 02	1.6729E + 04	2.1063E + 03
CDLOBA	3.7330E + 03	1.9094E + 02	4.3410E + 04	6.3077E + 03	5.7317E + 03	9.9061E + 02
HGWO	9.2565E + 03	5.6266E + 02	2.3789E + 04	**8.2974E + 02**	5.4537E + 03	2.0783E + 02
OBSCA	1.9805E + 04	2.3025E + 03	3.7320E + 04	1.9393E + 03	8.0478E + 03	6.3265E + 02
SMFO	2.3235E + 04	3.1999E + 03	4.6746E + 04	3.6419E + 03	1.0925E + 04	1.8727E + 03
CGSCA	1.6360E + 04	1.0295E + 03	3.5378E + 04	2.9080E + 03	7.0088E + 03	4.7392E + 02
RDWOA	4.0132E + 03	1.7287E + 02	2.4847E + 04	3.3510E + 03	4.3765E + 03	3.8047E + 02
m_SCA	9.7672E + 03	1.3584E + 03	2.1726E + 04	1.6597E + 03	4.7256E + 03	2.2278E + 02
BMWOA	2.3989E + 04	1.8122E + 03	4.7776E + 04	2.1187E + 03	1.0896E + 04	1.1218E + 03

	**F28**		**F29**		**F30**	
	**AVG**	**STD**	**AVG**	**STD**	**AVG**	**STD**

GACO	**3.9038E + 03**	2.2816E + 03	**6.4901E + 03**	9.3290E + 02	**9.1421E + 05**	**1.1131E + 06**
MOFOA	3.3321E + 04	5.6811E + 02	1.0369E + 06	2.1002E + 05	4.8865E + 10	3.6363E + 09
CDLOBA	6.5968E + 03	4.3899E + 03	1.2923E + 04	2.2381E + 03	7.6047E + 06	8.2526E + 06
HGWO	1.1358E + 04	8.2582E + 02	1.5530E + 04	**8.6072E + 02**	4.3779E + 09	1.0478E + 09
OBSCA	2.5167E + 04	1.9027E + 03	2.5075E + 04	5.6381E + 03	1.1665E + 10	2.4836E + 09
SMFO	2.5277E + 04	2.2267E + 03	1.1595E + 05	1.2348E + 05	2.2785E + 10	7.3377E + 09
CGSCA	2.1191E + 04	1.2692E + 03	1.9650E + 04	2.6334E + 03	7.8347E + 09	1.4230E + 09
RDWOA	4.1840E + 03	**2.3454E + 02**	1.0631E + 04	9.9180E + 02	5.1192E + 07	2.6411E + 07
m_SCA	1.3788E + 04	1.5582E + 03	1.0324E + 04	9.5652E + 02	1.9429E + 09	1.1864E + 09
BMWOA	2.6941E + 04	1.3195E + 03	2.3134E + 05	1.6466E + 05	3.1427E + 10	4.5072E + 09

In order to further analyze the performance of GACO on 30 benchmark functions, the ranking of all algorithms on each function is given in Table 9, where the advantage of GACO is clearly demonstrated, both on individual functions and overall performance is far better than other similar algorithms. The advanced performance of GACO is further verified by the Wilcoxon signed-rank test, which shows that GACO outperforms other algorithms on at least 23 benchmark functions.

**TABLE 9:** The ranking of all algorithms on each function and the Wilcoxon signed-rank test result.

F	GACO	MOFOA	CDLOBA	HGWO	OBSCA	SMFO	CGSCA	RDWOA	m_SCA	BMWOA
F1	1	10	2	4	7	8	6	3	5	9
F2	1	8	2	4	7	10	6	3	5	9
F3	4	5	9	6	8	7	3	2	1	10
F4	1	10	2	4	7	8	6	3	5	9
F5	1	9	7	4	6	8	5	3	2	10
F6	1	10	5	4	7	8	6	3	2	9
F7	1	8	10	2	6	7	5	4	3	9
F8	1	10	8	4	6	7	5	3	2	9
F9	1	9	4	5	8	6	7	2	3	10
F10	6	9	1	4	5	8	7	3	2	10
F11	1	8	2	7	6	9	5	3	4	10
F12	2	10	1	5	7	8	6	3	4	9
F13	1	10	2	5	7	8	6	3	4	9
F14	2	10	1	5	7	8	6	3	4	9
F15	1	10	3	5	7	8	6	2	4	9
F16	1	10	3	5	7	8	6	4	2	9
F17	1	10	4	5	7	8	6	3	2	9
F18	3	10	1	5	7	8	6	2	4	9
F19	1	10	3	5	7	8	6	2	4	9
F20	3	10	4	5	6	7	8	2	1	9
F21	1	10	7	3	5	8	6	4	2	9
F22	6	10	1	2	5	7	8	4	3	9
F23	1	10	7	4	6	9	5	3	2	8
F24	1	10	7	4	6	9	5	2	3	8
F25	1	10	2	4	7	8	6	3	5	9
F26	1	10	7	3	6	8	5	4	2	9
F27	1	10	5	4	7	9	6	2	3	8
F28	1	10	3	4	7	8	6	2	5	9
F29	1	10	4	5	7	8	6	3	2	9
F30	1	10	2	5	7	8	6	3	4	9
± / =	∼	29/0/1	24/5/1	27/2/1	27/2/1	28/0/2	27/1/2	23/4/3	25/4/1	30/0/0
Mean	1.63	9.53	3.97	4.37	6.60	7.97	5.87	2.87	3.13	9.07
Rank	1	10	4	5	7	8	6	2	3	9

**FIGURE 5:**
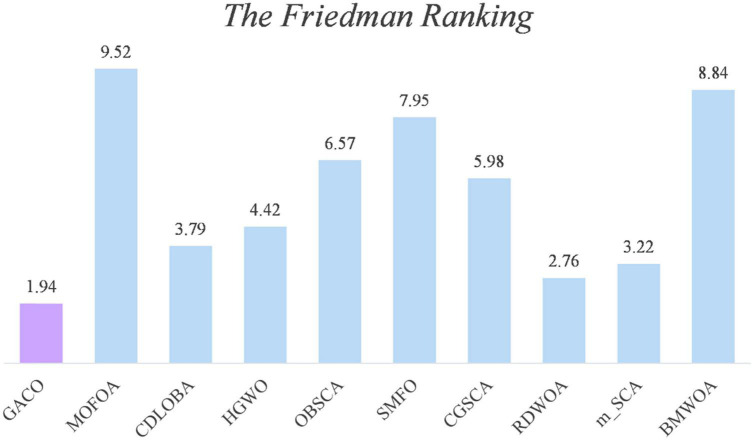
The Friedman ranking results for each algorithm.

**FIGURE 6:**
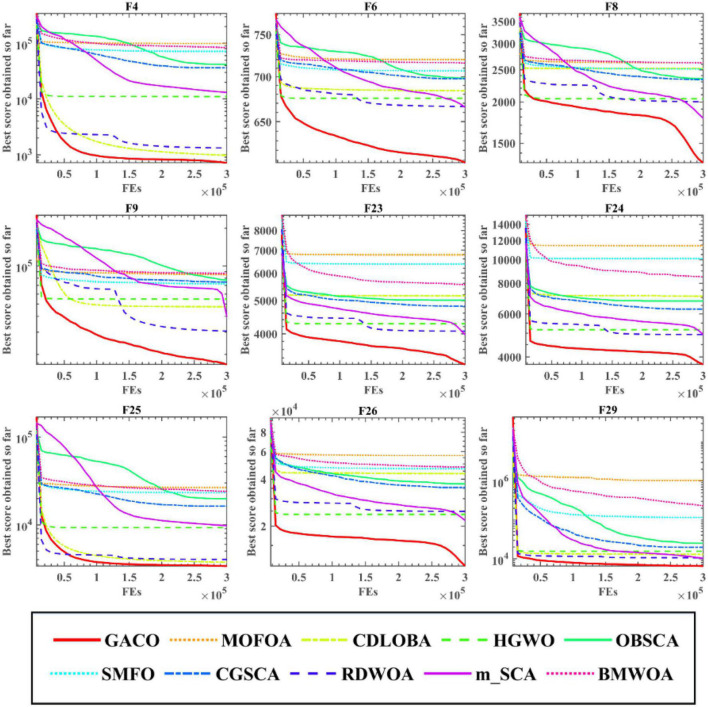
The convergence curves of all algorithms on some functions.

Next, the Friedman test analysis was further used to analyze the performance of the algorithms, and Figure 5 gives the Friedman ranking results for each algorithm. By observing the ranking results, it can be found that GACO ranks No. 1 with 1.94, followed by RDWOA in second place with 2.76, which effectively shows that GACO outperforms RDWOA, and likewise shows that GACO is better than other similar algorithms. Finally, the convergence curves of all algorithms on some functions are given in Figure 6, which clearly shows that GACO has a greater advantage in convergence performance than other similar variants of the algorithm.

Therefore, the core performance of GACO is further demonstrated by the comparison experiments between GACO and advanced variant algorithms, effectively showing that GACO is an excellent swarm optimization algorithm so that it can applied more fields, such as recommender system (Li et al., 2014, 2017), information retrieval services (Wu Z. et al., 2020; Wu et al., 2021), microgrids planning (Cao et al., 2021), clustering of cancer attributed networks (Gao et al., 2021; Wu and Ma, 2022), drug discovery (Zhu et al., 2018; Li Y. et al., 2020), disease identification and diagnosis (Su et al., 2019; Tian et al., 2020), image denoising (Zhang et al., 2020), tensor completion (Wang W. et al., 2022), colorectal polyp region extraction (Hu et al., 2022), drug repositioning (Cai et al., 2021), smart contract vulnerability detection (Zhang L. et al., 2022), human activity recognition (Qiu et al., 2022), structured sparsity optimization (Zhang X. et al., 2022), and medical data processing (Guo et al., 2022).

### 5.2 Feature selection experiments

In this subsection, the proposed bGACO-SVM is mainly applied to a variety of feature selection problems, mainly including tests on the public dataset, and tests on the TBPE dataset, as a way to show that it has good application capabilities.

#### 5.2.1 Experimental setup

In this subsection, firstly, bGACO is experimented with some similar methods on 11 public datasets, where the specific datasets involved are shown in Table 10. Then, bGACO is similarly compared with similar algorithms on the TBPE dataset, where the relevant description of the data is given in section “2 Data analysis.” In order to effectively illustrate the role of bGACO on TBPE, experiments comparing GACO with five very common machine learning algorithms are also performed. In order to ensure the reliability of the experiments, all experiments were conducted in the same environment as the benchmark function experiments, where some important parameters of the algorithms involved in the comparison are set using their settings in the original studies.

**TABLE 10:** Description of UCI datasets.

Datasets	Samples	Features	Classes
BreastEW	569	31	2
wdbc	569	31	2
JPNdata	152	11	2
IonosphereEW	351	35	2
CongressEW	435	17	2
SonarEW	208	61	2
Breastcancer	699	10	2
Heart	270	14	2
HeartEW	270	14	2
Vote	101	17	2
Wielaw	240	31	2

Accuracy, specificity, precision, the Mathews correlation coefficient (MCC), and the F-measure are the metrics that are used in order to assess how well the model performs in response to the outcomes of the experiments that were carried out. Accuracy refers to the proportion of cases correctly classified by the model, both in terms of true positives and true negatives. When the accuracy rate is higher, it suggests that a greater percentage of the samples have been accurately predicted. The term “specificity” refers to the percentage of “positive negatives” that are correctly classified by the model in “negative occurrences.” A lower rate of incorrect categorization is associated with a better specificity. The term “precision” refers to the likelihood that a given sample is positive out of the total number of samples for which a positive result is anticipated. When the precision is greater, it means that the forecast of affirmative instances is more precise. The MCC score provides insight into the model’s dependability. A more accurate forecast of the topic is indicated by an MCC that is closer to the value 1. A classifier may be evaluated in its entirety using the F-measure. When the F-measure is greater, it implies that the results of the classification are more in line with predictions. The definitions of the evaluation metrics may be found in Eqs (16)–(20).


(16) $$A\,c\,c\,u\,r\,a\,c\,y=\frac{T\,P+T\,N}{T\,P+F\,P+F\,N+T\,N}$$

(17) $$S\,p\,e\,c\,i\,f\,i\,c\,i\,t\,y=\frac{T\,N}{T\,N+F\,P}$$

(18) $$P\,r\,e\,c\,i\,s\,i\,o\,n=\frac{T\,P}{T\,P+F\,P}$$

(19) $$M\,C\,C=\frac{T\,P\times T\,N-F\,P\times F\,N}{\sqrt{\left(T\,P+F\,P\right)\times \left(T\,P+F\,N\right)\times \left(T\,N+F\,P\right)\times \left(T\,N+F\,N\right)}}$$

(20) $$F-m\,e\,a\,s\,u\,r\,e=\frac{T\,P}{T\,P+\frac{F\,N+F\,P}{2}}$$

where TP represents the number of occurrences of a true positive, TN represents the number of instances of a true negative, FN represents the number of instances of a false negative, and FP represents the number of instances of a false positive.

#### 5.2.2 Public dataset experiment

The overall predictive potential of the bGACO-SVM is illustrated in this portion of the article. On various datasets from the University of California, Irvine (UCI),^1^ which is an open-source dataset suitable for the direction of pattern recognition and machine learning, and many scholars choose to use the dataset on UCI to verify the correctness of their proposed algorithms. The bGACO-SVM feature selection framework is put up against 12 different standard approaches, which include bACOR, bCSO, bWOA, bGWO, bHHO, bJAYA, bPSO, bSCA, bSMA, bSSA, bDE, and bFA.

Table 11 compares the accuracy of 13 different algorithms applied to various datasets from the University of California, Irvine, and finds that the bGACO-based technique achieves the highest average accuracy over 20 separate tests, where the best results are bolded in each column. The bGACO-based technique has an accuracy of 99.48, 99.83, 99.33, 100.00, 99.77, 100.00, 99.43, 97.04, 95.93, 100.00, and 92.10%, respectively, for each of the nine datasets. Only the technique that was based on bSMA had a standard deviation that was significantly lower than the others in the BreastEW dataset. In the Breastcancer dataset, only the bJAYA-based technique had a standard deviation that was significantly lower than the others. Only the approach that was based on bHHO had a standard deviation that was much lower than the others in the heart dataset. In addition, the experimental outcomes of the bGACO-based technique are more consistent when applied to different datasets. According to the findings of the aforementioned investigation, the bGACO-SVM feature selection approach exceeds any and all other methods with regard to the accuracy and consistency of its predictions.

**TABLE 11:** Accuracy value on UCI datasets.

Methods	BreastEW		wdbc		JPNdata		IonosphereEW	
	Mean	Std	Mean	Std	Mean	Std	Mean	Std
bGACO	**99.48%**	0.0117	**99.83%**	**0.0055**	**99.33%**	**0.0211**	**100.00%**	**0.0000**
bACOR	99.12%	0.0124	99.82%	0.0056	96.83%	0.0445	99.71%	0.0093
bCSO	99.12%	0.0093	99.48%	0.0084	98.67%	0.0281	**100.00%**	**0.0000**
bWOA	99.30%	0.0169	99.65%	0.0075	96.79%	0.0339	99.71%	0.0090
bGWO	99.30%	0.0122	99.65%	0.0073	98.71%	0.0272	**100.00%**	**0.0000**
bHHO	99.29%	0.0149	99.65%	0.0075	98.13%	0.0422	**100.00%**	**0.0000**
bJAYA	99.12%	0.0124	**99.83%**	**0.0055**	96.74%	0.0453	**100.00%**	**0.0000**
bPSO	99.12%	0.0092	99.47%	0.0085	96.61%	0.0491	**100.00%**	**0.0000**
bSCA	99.30%	0.0121	99.48%	0.0084	96.75%	0.0343	99.71%	0.0093
bSMA	99.30%	**0.0091**	99.65%	0.0074	97.33%	0.0346	**100.00%**	**0.0000**
bSSA	99.13%	0.0149	99.47%	0.0085	97.42%	0.0334	**100.00%**	**0.0000**
bDE	98.24%	0.0186	98.96%	0.0146	93.71%	0.0722	98.56%	0.0203
bFA	99.12%	0.0124	99.48%	0.0084	96.08%	0.0631	**100.00%**	**0.0000**

**Methods**	**CongressEW**		**SonarEW**		**Breastcancer**		**heart**	
	**Mean**	**Std**	**Mean**	**Std**	**Mean**	**Std**	**Mean**	**Std**

bGACO	**99.77%**	**0.0074**	**100.00%**	**0.0000**	**99.43%**	0.0138	**97.04%**	0.0292
bACOR	99.08%	0.0119	98.57%	0.0321	98.71%	0.0142	95.19%	0.0305
bCSO	99.55%	0.0096	99.00%	0.0316	98.86%	0.0131	95.19%	0.0495
bWOA	98.86%	0.0221	98.55%	0.0234	98.86%	0.0113	94.44%	0.0436
bGWO	99.07%	0.0163	99.50%	0.0158	99.28%	0.0101	94.82%	0.0358
bHHO	99.31%	0.0111	99.05%	0.0301	98.86%	0.0113	93.70%	**0.0250**
bJAYA	99.08%	0.0119	99.52%	0.0151	98.71%	**0.0082**	94.82%	0.0312
bPSO	99.09%	0.0159	98.55%	0.0234	99.14%	0.0121	94.82%	0.0435
bSCA	98.85%	0.0162	99.52%	0.0151	99.00%	0.0118	96.30%	0.0462
bSMA	99.08%	0.0160	99.52%	0.0151	99.00%	0.0118	94.82%	0.0259
bSSA	99.31%	0.0112	99.02%	0.0206	99.14%	0.0121	95.93%	0.0408
bDE	97.49%	0.0227	94.69%	0.0616	97.57%	0.0096	86.30%	0.0631
bFA	99.31%	0.0112	99.05%	0.0201	99.00%	0.0118	95.56%	0.0292

**Methods**	**HeartEW**		**Vote**		**Wielaw**			
	**Mean**	**Std**	**Mean**	**Std**	**Mean**	**Std**		

bGACO	**95.93%**	**0.0273**	**100.00%**	**0.0000**	**92.10%**	**0.0305**		
bACOR	94.82%	0.0358	99.00%	0.0225	90.81%	0.0826		
bCSO	94.44%	0.0501	99.33%	0.0141	91.21%	0.0624		
bWOA	93.33%	0.0340	98.69%	0.0228	90.44%	0.0577		
bGWO	95.56%	0.0625	98.68%	0.0232	91.21%	0.0379		
bHHO	94.07%	0.0358	98.66%	0.0234	90.04%	0.0835		
bJAYA	95.56%	0.0383	98.99%	0.0163	90.39%	0.0350		
bPSO	93.70%	0.0429	99.34%	0.0138	89.13%	0.0417		
bSCA	95.56%	0.0340	99.02%	0.0219	90.79%	0.0594		
bSMA	94.07%	0.0435	99.67%	0.0105	90.81%	0.0436		
bSSA	94.82%	0.0358	98.65%	0.0238	91.28%	0.0594		
bDE	87.41%	0.0468	96.63%	0.0317	84.56%	0.0636		
bFA	94.44%	0.0400	99.68%	0.0102	90.00%	0.0562		

The specificity ratings for each of the 10 algorithms are shown in Table 12. The best results are bolded in each column. It is clear from looking at the table that the bGACO-based technique yields mean values that are all equal to No 1. The typical results of Bgaco’s Specificity test may achieve values as high as 98.62, 100.00, 98.57, 100.00, 100.00, 100.00, 100.00, 100.00, 100.00, 100.00, 95.00, 95.00, 100.00, and 92.95%, respectively. Only for the BreastEW, JPNdata, heart, and HeartEW do the results of the standard deviation not show the greatest performance, but the prediction results of bGACO are similarly stable in all other cases. As a result, the bGACO-SVM has a smaller classification error in these comparing methodologies’ species.

**TABLE 12:** Specificity value on UCI datasets.

Methods	BreastEW		wdbc		JPNdata		IonosphereEW	
	Mean	Std	Mean	Std	Mean	Std	Mean	Std
bGACO	**98.62%**	0.0309	**100.00%**	**0.0000**	98.57%	0.0452	**100.00%**	**0.0000**
bACOR	98.10%	0.0333	**100.00%**	**0.0000**	95.00%	0.0874	99.17%	0.0264
bCSO	97.62%	0.0251	**100.00%**	**0.0000**	97.32%	0.0566	**100.00%**	**0.0000**
bWOA	98.57%	0.0321	**100.00%**	**0.0000**	95.89%	0.0663	99.17%	0.0264
bGWO	98.12%	0.0331	**100.00%**	**0.0000**	97.50%	0.0527	**100.00%**	**0.0000**
bHHO	98.10%	0.0402	**100.00%**	**0.0000**	**98.75%**	**0.0395**	**100.00%**	**0.0000**
bJAYA	98.12%	0.0331	**100.00%**	**0.0000**	97.32%	0.0566	**100.00%**	**0.0000**
bPSO	98.12%	0.0243	**100.00%**	**0.0000**	97.32%	0.0566	**100.00%**	**0.0000**
bSCA	98.14%	0.0322	**100.00%**	**0.0000**	97.50%	0.0527	99.17%	0.0264
bSMA	98.59%	**0.0227**	**100.00%**	**0.0000**	97.32%	0.0566	**100.00%**	**0.0000**
bSSA	97.64%	0.0403	**100.00%**	**0.0000**	94.82%	0.0671	**100.00%**	**0.0000**
bDE	96.67%	0.0392	99.72%	0.0088	92.50%	0.0874	95.90%	0.0585
bFA	98.59%	0.0319	**100.00%**	**0.0000**	96.25%	0.0844	**100.00%**	**0.0000**

**Methods**	**CongressEW**		**SonarEW**		**Breastcancer**		**heart**	
	**Mean**	**Std**	**Mean**	**Std**	**Mean**	**Std**	**Mean**	**Std**

bGACO	**100.00%**	**0.0000**	**100.00%**	**0.0000**	**100.00%**	**0.0000**	**95.00%**	0.0583
bACOR	98.82%	0.0248	99.09%	0.0287	98.77%	0.0199	91.67%	0.0680
bCSO	**100.00%**	**0.0000**	99.09%	0.0287	98.75%	0.0201	93.33%	0.1024
bWOA	98.82%	0.0372	99.09%	0.0287	99.17%	0.0264	92.50%	0.0615
bGWO	98.79%	0.0256	99.09%	0.0287	99.17%	0.0176	90.83%	0.0730
bHHO	**100.00%**	**0.0000**	99.09%	0.0287	98.75%	0.0201	91.67%	0.0786
bJAYA	99.41%	0.0186	**100.00%**	**0.0000**	99.17%	0.0176	94.17%	0.0791
bPSO	99.41%	0.0186	99.09%	0.0287	99.18%	0.0172	92.50%	0.0730
bSCA	99.38%	0.0198	**100.00%**	**0.0000**	98.77%	0.0199	94.17%	0.0686
bSMA	98.82%	0.0248	**100.00%**	**0.0000**	**100.00%**	**0.0000**	94.17%	0.0686
bSSA	**100.00%**	**0.0000**	99.09%	0.0287	98.75%	0.0281	93.33%	0.0766
bDE	98.24%	0.0284	95.46%	0.0643	97.52%	0.0290	80.83%	0.0791
bFA	**100.00%**	**0.0000**	99.09%	0.0287	98.35%	0.0290	92.50%	**0.0473**

**Methods**	**HeartEW**		**Vote**		**Wielaw**			
	**Mean**	**Std**	**Mean**	**Std**	**Mean**	**Std**		

bGACO	**95.00%**	0.0583	**100.00%**	**0.0000**	**92.95%**	**0.0440**		
bACOR	92.50%	0.0917	98.89%	0.0351	**92.95%**	0.0862		
bCSO	94.17%	0.0562	99.47%	0.0166	91.35%	0.0778		
bWOA	92.50%	0.0829	97.87%	0.0371	89.04%	0.0833		
bGWO	94.17%	0.1115	98.39%	0.0259	**92.95%**	0.0570		
bHHO	90.83%	0.0829	98.36%	0.0372	92.12%	0.0825		
bJAYA	**95.00%**	0.0583	98.92%	0.0228	92.24%	0.0726		
bPSO	88.33%	0.0978	98.95%	0.0222	88.97%	0.0777		
bSCA	92.50%	0.0615	99.47%	0.0166	88.27%	0.0835		
bSMA	90.83%	0.0829	**100.00%**	**0.0000**	92.12%	0.0741		
bSSA	**95.00%**	**0.0430**	97.81%	0.0386	**92.95%**	0.0692		
bDE	85.83%	0.0966	96.70%	0.0387	85.90%	0.1286		
bFA	94.17%	0.0791	99.47%	0.0166	89.94%	0.0728		

The mean as well as the standard deviation of the accuracy findings are shown in Table 13. The best results are bolded in each column. The bGACO-based technique achieved average outcomes of 99.20, 100.00, 98.89, 100.00, 100.00, 100.00, 100.00, 96.28, 96.28, 100.00, and 92.20%, respectively, when applied to all 11 datasets. Table 13 shows that the bGACO-based technique delivers the greatest and most consistent Precision results overall. These findings are based on an average of the measurements. Therefore, when compared to other techniques of prediction, bGACO-SVM has a higher level of accuracy when it comes to forecasting positive cases.

**TABLE 13:** Precision value on UCI datasets.

Methods	BreastEW		Wdbc		JPNdata		IonosphereEW	
	Mean	Std	Mean	Std	Mean	Std	Mean	Std
bGACO	**99.20%**	0.0178	**100.00%**	**0.0000**	**98.89%**	**0.0351**	**100.00%**	**0.0000**
bACOR	98.92%	0.0187	**100.00%**	**0.0000**	95.78%	0.0722	99.57%	0.0137
bCSO	98.64%	0.0143	**100.00%**	**0.0000**	97.64%	0.0499	**100.00%**	**0.0000**
bWOA	99.19%	0.0182	**100.00%**	**0.0000**	96.67%	0.0537	99.58%	0.0132
bGWO	98.93%	0.0185	**100.00%**	**0.0000**	97.64%	0.0499	**100.00%**	**0.0000**
bHHO	98.93%	0.0225	**100.00%**	**0.0000**	**98.89%**	**0.0351**	**100.00%**	**0.0000**
bJAYA	98.92%	0.0187	**100.00%**	**0.0000**	97.50%	0.0527	**100.00%**	**0.0000**
bPSO	98.91%	0.0141	**100.00%**	**0.0000**	97.32%	0.0566	**100.00%**	**0.0000**
bSCA	98.93%	0.0186	**100.00%**	**0.0000**	97.64%	0.0499	99.57%	0.0137
bSMA	99.18%	**0.0132**	**100.00%**	**0.0000**	97.78%	0.0468	**100.00%**	**0.0000**
bSSA	98.68%	0.0224	**100.00%**	**0.0000**	95.42%	0.0593	**100.00%**	**0.0000**
bDE	98.09%	0.0223	99.55%	0.0144	92.78%	0.0815	97.90%	0.0291
bFA	99.20%	0.0179	**100.00%**	**0.0000**	96.57%	0.0735	**100.00%**	**0.0000**

**Methods**	**CongressEW**		**SonarEW**		**Breastcancer**		**heart**	
	**Mean**	**Std**	**Mean**	**Std**	**Mean**	**Std**	**Mean**	**Std**

bGACO	**100.00%**	**0.0000**	**100.00%**	**0.0000**	**100.00%**	**0.0000**	**96.28%**	0.0424
bACOR	99.27%	0.0153	99.00%	0.0316	99.36%	0.0104	93.89%	0.0481
bCSO	**100.00%**	**0.0000**	98.89%	0.0351	99.35%	0.0104	95.27%	0.0660
bWOA	99.29%	0.0226	99.00%	0.0316	99.58%	0.0132	94.27%	0.0454
bGWO	99.27%	0.0153	99.00%	0.0316	99.57%	0.0092	93.30%	0.0522
bHHO	**100.00%**	**0.0000**	99.00%	0.0316	99.35%	0.0104	93.93%	0.0564
bJAYA	99.63%	0.0117	**100.00%**	**0.0000**	99.57%	0.0092	95.75%	0.0542
bPSO	99.63%	0.0117	99.09%	0.0287	99.57%	0.0091	94.41%	0.0498
bSCA	99.64%	0.0113	**100.00%**	**0.0000**	99.35%	0.0105	95.58%	0.0516
bSMA	99.26%	0.0156	**100.00%**	**0.0000**	**100.00%**	**0.0000**	95.69%	0.0493
bSSA	**100.00%**	**0.0000**	99.00%	0.0316	99.37%	0.0141	95.15%	0.0553
bDE	98.89%	0.0179	95.09%	0.0701	98.71%	0.0150	85.65%	0.0528
bFA	**100.00%**	**0.0000**	99.09%	0.0287	99.15%	0.0147	94.32%	**0.0343**

**Methods**	**HeartEW**		**Vote**		**Wielaw**			
	**Mean**	**Std**	**Mean**	**Std**	**Mean**	**Std**		

bGACO	**96.28%**	0.0424	**100.00%**	**0.0000**	92.20%	**0.0481**		
bACOR	94.69%	0.0629	98.57%	0.0452	92.11%	0.0955		
bCSO	95.37%	0.0449	99.23%	0.0243	90.75%	0.0819		
bWOA	94.48%	0.0588	96.97%	0.0517	88.58%	0.0817		
bGWO	95.90%	0.0743	97.55%	0.0396	**92.30%**	0.0605		
bHHO	93.35%	0.0564	97.74%	0.0497	91.05%	0.0921		
bJAYA	96.19%	0.0431	98.40%	0.0338	91.71%	0.0743		
bPSO	91.75%	0.0630	98.40%	0.0338	88.50%	0.0753		
bSCA	94.40%	0.0440	99.17%	0.0264	88.02%	0.0832		
bSMA	93.28%	0.0571	**100.00%**	**0.0000**	91.64%	0.0791		
bSSA	96.16%	**0.0332**	96.92%	0.0538	92.09%	0.0733		
bDE	89.20%	0.0701	95.26%	0.0547	85.49%	0.1208		
bFA	95.74%	0.0542	99.23%	0.0243	89.03%	0.0765		

The mean and standard deviation of the MCC are shown in Table 14. The best results are bolded in each column. The approach that was suggested in this article achieved mean outcomes of 0.9891, 0.9963, 0.9873, 1.0000, 0.9953, 1.0000, 0.9880, 0.9417, 0.9207, 1.0000, and 0.8439, respectively, in the MCC. The comparative findings are shown in Table 14, and they reveal that the bGACO-based technique is capable of demonstrating superior performance and more reliable outcomes, where the best results are bolded in each column. As a result, the bGACO-SVM that was presented is an improved method for making predictions using the target dataset.

**TABLE 14:** MCC value on UCI datasets.

Methods	BreastEW		Wdbc		JPNdata		IonosphereEW	
	Mean	Std	Mean	Std	Mean	Std	Mean	Std
bGACO	**0.9891**	0.0245	**0.9963**	**0.0118**	**0.9873**	**0.0402**	**1.0000**	**0.0000**
bACOR	0.9815	0.0262	0.9962	0.0119	0.9413	0.0816	0.9936	0.0201
Bcso	0.9813	0.0197	0.9889	0.0179	0.9748	0.0532	**1.0000**	**0.0000**
bWOA	0.9849	0.0365	0.9925	0.0158	0.9392	0.0642	0.9937	0.0198
bGWO	0.9852	0.0259	0.9926	0.0155	0.9757	0.0513	**1.0000**	**0.0000**
bHHO	0.9851	0.0314	0.9925	0.0158	0.9657	0.0767	**1.0000**	**0.0000**
bJAYA	0.9815	0.0262	**0.9963**	**0.0118**	0.9373	0.0886	**1.0000**	**0.0000**
bPSO	0.9815	0.0195	0.9888	0.0181	0.9346	0.0967	**1.0000**	**0.0000**
bSCA	0.9853	0.0255	0.9889	0.0179	0.9387	0.0647	0.9936	0.0201
bSMA	0.9852	**0.0192**	0.9925	0.0158	0.9494	0.0655	**1.0000**	**0.0000**
bSSA	0.9815	0.0315	0.9888	0.0180	0.9512	0.0631	**1.0000**	**0.0000**
bDE	0.9625	0.0398	0.9780	0.0308	0.8755	0.1434	0.9688	0.0441
bFA	0.9816	0.0261	0.9889	0.0179	0.9263	0.1191	**1.0000**	**0.0000**

**Methods**	**CongressEW**		**SonarEW**		**Breastcancer**		**heart**	
	**Mean**	**Std**	**Mean**	**Std**	**Mean**	**Std**	**Mean**	**Std**

bGACO	**0.9953**	**0.0149**	**1.0000**	**0.0000**	**0.9880**	0.0286	**0.9417**	0.0577
bACOR	0.9811	0.0244	0.9717	0.0640	0.9724	0.0300	0.9055	0.0600
bCSO	0.9907	0.0195	0.9798	0.0639	0.9750	0.0287	0.9064	0.0941
bWOA	0.9761	0.0467	0.9721	0.0449	0.9753	0.0243	0.8900	0.0873
bGWO	0.9812	0.0325	0.9905	0.0302	0.9844	0.0223	0.8980	0.0712
bHHO	0.9859	0.0227	0.9809	0.0604	0.9749	0.0249	0.8771	**0.0503**
bJAYA	0.9811	0.0245	0.9908	0.0290	0.9719	**0.0180**	0.8991	0.0599
bPSO	0.9810	0.0334	0.9720	0.0451	0.9815	0.0259	0.8985	0.0849
bSCA	0.9767	0.0325	0.9908	0.0290	0.9781	0.0258	0.9255	0.0934
bSMA	0.9808	0.0335	0.9908	0.0290	0.9786	0.0250	0.8989	0.0507
bSSA	0.9859	0.0228	0.9813	0.0395	0.9814	0.0261	0.9206	0.0801
bDE	0.9490	0.0452	0.8960	0.1227	0.9468	0.0216	0.7254	0.1280
bFA	0.9859	0.0228	0.9817	0.0385	0.9783	0.0254	0.9116	0.0586

**Methods**	**HeartEW**		**Vote**		**Wielaw**			
	**Mean**	**Std**	**Mean**	**Std**	**Mean**	**Std**		

bGACO	**0.9207**	**0.0530**	**1.0000**	**0.0000**	**0.8439**	**0.0613**		
bACOR	0.9001	0.0682	0.9802	0.0438	0.8230	0.1648		
bCSO	0.8895	0.1008	0.9863	0.0289	0.8279	0.1239		
bWOA	0.8695	0.0685	0.9741	0.0445	0.8129	0.1149		
bGWO	0.9137	0.1214	0.9725	0.0491	0.8297	0.0749		
bHHO	0.8845	0.0694	0.9733	0.0458	0.8038	0.1676		
bJAYA	0.9131	0.0752	0.9794	0.0333	0.8135	0.0661		
bPSO	0.8775	0.0817	0.9867	0.0280	0.7881	0.0832		
bSCA	0.9119	0.0676	0.9796	0.0459	0.8210	0.1167		
bSMA	0.8835	0.0856	0.9932	0.0215	0.8225	0.0877		
bSSA	0.9004	0.0671	0.9736	0.0463	0.8296	0.1188		
bDE	0.7492	0.0960	0.9331	0.0624	0.7023	0.1276		
bFA	0.8936	0.0766	0.9935	0.0205	0.8034	0.1111		

Table 15 displays the mean and standard deviation of the F-measure. The best results are bolded in each column. It can be seen that the mean standards of bGACO-based method reached 99.59, 99.76, 99.41, 100.00, 99.80, 100.00, 99.55, 97.40, 96.34, 100.00, and 91.49%, respectively. The bGACO-based method is the most stable among the experimental results of wdbc, JPNdata, IonosphereEW, CongressEW, SonarEW, HeartEW, Vote, and Wielaw. According to the analysis of Table 15, the experimental data shows that the bGACO-SVM technique is a more effective classification method than the others.

**TABLE 15:** F-measure value on UCI datasets.

Methods	BreastEW		wdbc		JPNdata		IonosphereEW	
	Mean	Std	Mean	Std	Mean	Std	Mean	Std
bGACO	**99.59%**	0.0091	**99.76%**	**0.0077**	**99.41%**	**0.0186**	**100.00%**	**0.0000**
bACOR	99.31%	0.0097	**99.76%**	**0.0077**	97.05%	0.0408	99.78%	0.0070
bCSO	99.31%	0.0073	99.28%	0.0116	98.75%	0.0265	**100.00%**	**0.0000**
bWOA	99.45%	0.0132	99.51%	0.0103	96.90%	0.0328	99.79%	0.0067
bGWO	99.46%	0.0095	99.52%	0.0100	98.75%	0.0265	**100.00%**	**0.0000**
bHHO	99.45%	0.0116	99.51%	0.0103	97.98%	0.0469	**100.00%**	**0.0000**
bJAYA	99.31%	0.0097	**99.76%**	**0.0077**	96.75%	0.0452	**100.00%**	**0.0000**
bPSO	99.30%	0.0073	99.27%	0.0118	96.57%	0.0494	**100.00%**	**0.0000**
bSCA	99.45%	0.0095	99.28%	0.0116	96.64%	0.0357	99.78%	0.0070
bSMA	99.44%	**0.0072**	99.51%	0.0103	97.29%	0.0356	**100.00%**	**0.0000**
bSSA	99.32%	0.0115	99.28%	0.0116	97.57%	0.0315	**100.00%**	**0.0000**
bDE	98.62%	0.0145	98.58%	0.0198	93.82%	0.0701	98.92%	0.0151
bFA	99.31%	0.0097	99.28%	0.0116	95.79%	0.0690	**100.00%**	**0.0000**

**Methods**	**CongressEW**		**SonarEW**		**Breastcancer**		**heart**	
	**Mean**	**Std**	**Mean**	**Std**	**Mean**	**Std**	**Mean**	**Std**

bGACO	**99.80%**	**0.0062**	**100.00%**	**0.0000**	**99.55%**	0.0109	**97.40%**	0.0256
bACOR	99.25%	0.0098	98.47%	0.0341	99.01%	0.0111	95.80%	0.0264
bCSO	99.62%	0.0080	98.89%	0.0351	99.13%	0.0101	95.79%	0.0416
bWOA	99.08%	0.0178	98.42%	0.0254	99.12%	0.0086	95.03%	0.0398
bGWO	99.23%	0.0137	99.47%	0.0166	99.45%	0.0077	95.50%	0.0308
bHHO	99.43%	0.0091	99.00%	0.0316	99.12%	0.0086	94.42%	**0.0213**
bJAYA	99.24%	0.0098	99.47%	0.0166	99.01%	**0.0063**	95.36%	0.0267
bPSO	99.25%	0.0130	98.41%	0.0258	99.34%	0.0094	95.39%	0.0391
bSCA	99.04%	0.0136	99.47%	0.0166	99.23%	0.0090	96.75%	0.0404
bSMA	99.25%	0.0130	99.47%	0.0166	99.22%	0.0092	95.35%	0.0230
bSSA	99.43%	0.0092	98.95%	0.0222	99.34%	0.0093	96.42%	0.0358
bDE	97.91%	0.0193	94.22%	0.0667	98.14%	0.0072	87.96%	0.0566
bFA	99.43%	0.0092	99.00%	0.0212	99.24%	0.0090	96.09%	0.0260

**Methods**	**HeartEW**		**Vote**		**Wielaw**			
	**Mean**	**Std**	**Mean**	**Std**	**Mean**	**Std**		

bGACO	**96.34%**	**0.0248**	**100.00%**	**0.0000**	**91.49%**	**0.0322**		
bACOR	95.45%	0.0304	98.76%	0.0271	89.64%	0.0970		
bCSO	94.94%	0.0459	99.12%	0.0186	90.63%	0.0666		
bWOA	94.03%	0.0287	98.40%	0.0276	90.08%	0.0582		
bGWO	96.11%	0.0538	98.29%	0.0308	90.34%	0.0442		
bHHO	94.81%	0.0311	98.32%	0.0284	89.05%	0.0926		
bJAYA	95.97%	0.0352	98.69%	0.0212	89.53%	0.0371		
bPSO	94.63%	0.0348	99.17%	0.0176	88.38%	0.0454		
bSCA	96.11%	0.0299	98.73%	0.0283	90.59%	0.0599		
bSMA	94.81%	0.0380	99.57%	0.0137	89.95%	0.0493		
bSSA	95.18%	0.0355	98.37%	0.0288	90.40%	0.0688		
bDE	88.71%	0.0411	95.63%	0.0437	83.43%	0.0651		
bFA	94.95%	0.0363	99.60%	0.0126	89.39%	0.0593		

In this part, comparison experiments are conducted using the UCI dataset. Based on the results of these tests, it is clear that bGACO-SVM has dependable and outstanding prediction performance. Based on the results of the experiments including accuracy, specificity, precision, MCC, and F-measure, it would seem that bGACO-SVM is successful in achieving the target it was intended for.

#### 5.2.3 TBPE dataset experiment

Comparisons are made between bGACO-SVM and 13 other comparable techniques, also including bACOR, bCSO, bWOA, bGWO, bHHO, bJAYA, bMFO, bPSO, bSCA, bSMA, bSSA, bDE, and bFA. This is done to prove that bGACO-SVM is extremely competitive when compared to other methods of a similar kind.

**FIGURE 7:**
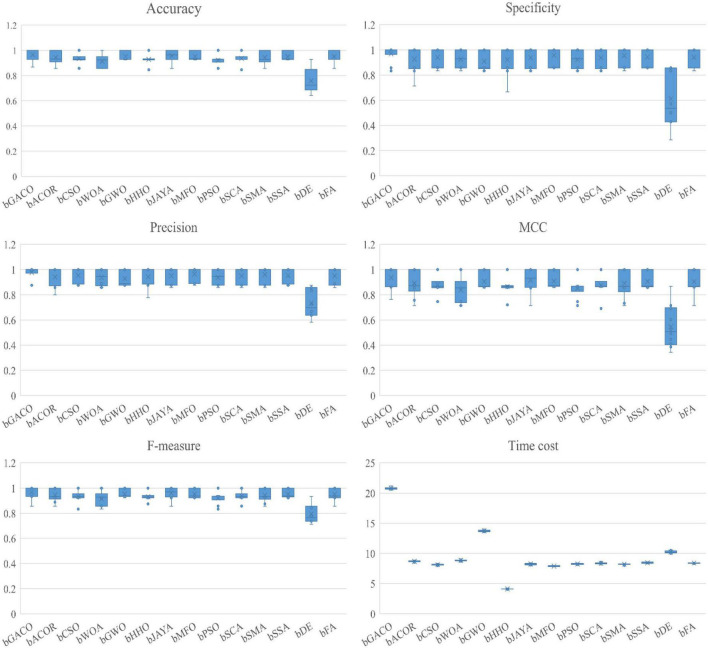
Boxplot of the performance of 10 methods in six metrics.

The findings of the six assessment criteria are broken down and averaged in Table 16. It is clear to observe that the bGACO-SVM has an accuracy of 96.57%, specificity of 96.91%, precision of 97.64%, MCC of 0.9366, and an F-measure of 96.65%, respectively. Despite the fact that the bGACO-SVM algorithm requires a considerable amount of time. Along with the large gain in classification accuracy, a certain amount of additional time consumption is unavoidable; nevertheless, we have the ability to make up for this shortfall by using methods for parallel computing or boosting the computational capacity of computing equipment. The 10 iterations of the boxplot for the 10 different algorithms are shown in Figure 7. The maximum, median, and lowest values shown in the figure demonstrate that the experimental results of bGACO-SVM are both outstanding and consistent. The good classification results that bGACO-SVM produces are not the product of a few lucky tests; rather, they are the consequence of the system’s consistency and its outstanding classification performance.

**TABLE 16:** Average values of 10 methods in the six metrics.

Algorithms	Accuracy		Specificity		Precision	
	Value	Rank	Value	Rank	Value	Rank
bGACO	96.57%	1	96.91%	1	97.64%	1
bACOR	94.37%	7	92.62%	9	94.07%	10
bCSO	93.56%	9	94.05%	5	95.28%	4
bWOA	91.41%	13	92.62%	9	93.67%	12
bGWO	94.99%	5	90.95%	13	92.78%	13
bHHO	92.78%	11	92.14%	12	94.31%	9
bJAYA	95.66%	2	93.81%	7	94.96%	6
bMFO	95.09%	3	95.71%	2	96.53%	2
bPSO	92.14%	12	92.38%	11	93.71%	11
bSCA	93.51%	10	93.81%	7	94.96%	6
bSMA	94.33%	8	95.48%	3	96.07%	3
bSSA	94.99%	6	94.29%	4	95.28%	4
bDE	75.81%	14	61.19%	14	73.39%	14
bFA	95.00%	4	94.05%	5	94.96%	6

**Algorithms**	**MCC**		**F-measure**		**Time cost**	
	**Value**	**Rank**	**Value**	**Rank**	**Value**	**Rank**

bGACO	0.9366	1	96.65%	1	20.7936	14
bACOR	0.8931	7	94.69%	7	8.6635	10
bCSO	0.8800	9	93.52%	10	8.1199	3
bWOA	0.8381	13	91.44%	13	8.8293	11
bGWO	0.9052	5	95.39%	3	13.7221	13
bHHO	0.8650	11	93.16%	11	4.1057	1
bJAYA	0.9161	2	95.88%	2	8.2238	5
bMFO	0.9076	3	95.18%	5	7.8886	2
bPSO	0.8513	12	92.21%	12	8.237	6
bSCA	0.8756	10	93.68%	9	8.3403	7
bSMA	0.8898	8	94.45%	8	8.1968	4
bSSA	0.9058	4	95.29%	4	8.4339	9
bDE	0.5471	14	79.56%	14	10.2055	12
bFA	0.9039	6	95.11%	6	8.3914	8

**FIGURE 8:**
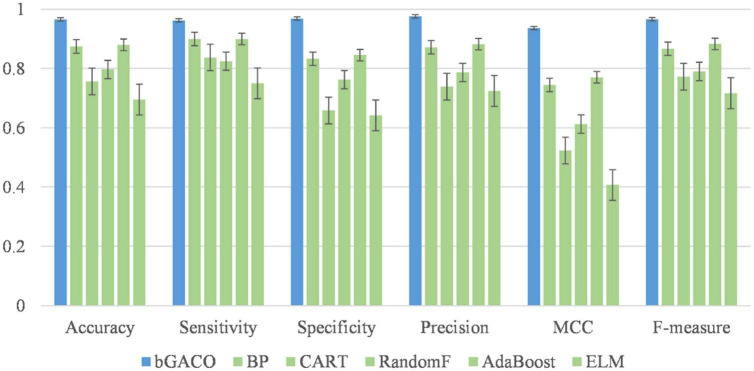
Comparison of bGACO-SVM with well-known classifiers.

In this part, the bGACO-SVM algorithm is evaluated with a number of other well-known classifiers. It is clear from looking at Figure 8 that the classification approach that performs the best out of the six options is the bGACO-SVM, followed by the BP neural network. The results of the Extreme Learning Machine (ELM) for forecasting TBPE are disappointing. This suggests that classification models based on bGACO and SVM can make up for the deficiency of single classifier in classification and provide classification results with a greater level of accuracy.

**FIGURE 9:**
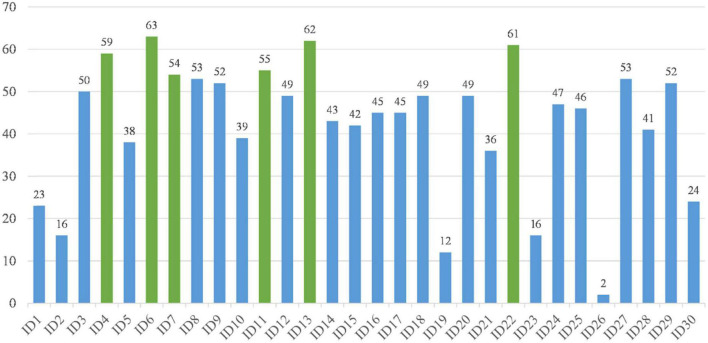
The number of times each feature was selected in 100 experiments.

The above experimental study allows us to draw the conclusion that bGACO-SVM has the potential to produce a feature subset for the TBPE dataset that has superior outcomes. We increased the number of runs to a hundred so that we could assess whether or not the best feature subset that was chosen is relevant for medical diagnosis. The frequency with which each characteristic occurred served as a reliable indicator of the significance of those characteristics in terms of clinical diagnosis. Figure 9 shows the number of occurrences of the overall features. The main characteristics that affect TEPB are temperature, age, white blood cell, percentage of monocyte, absolute value of eosinophils, mean corpuscular hemoglobin, where age (ID6) is selected 63 times, absolute value of eosinophils (ID13) is selected 62 times, mean corpuscular hemoglobin (ID22) is selected 61 times, serum temperature (ID4) is selected 59 times, percentage of monocyte (ID11) is selected 55 times, and white blood cell (ID7) is selected 54 times. These features are not negligible in the TEPB forecast.

## 6 Discussion

It is suspected that patients with TBPE need rapid accurate diagnosis and immediate treatment, otherwise it can cause tuberculous abscess, thickening of pleural membrane, thoracic malformations, and other adverse consequences. This study by the method of GACO, only used the patients’ general clinical condition and routine blood test results, set up effective diagnosis model to distinguish the TBPE and non-TBPE. The model diagnosis accuracy ACC reached 96.57%, MCC was 0.9366, F-measure and specificity of 96.65 and 96.91%, respectively.

In this experiment, we acquired a relatively high diagnostic accuracy with the methods of combination. We also carried out the statistics and found several of the characteristics had relatively high frequency, which is consistent with the actual situation of clinical medicine. Among the high frequency characteristics were body temperature, WBC count, absolute value of eosinophils, age, percentage of monocytes, and mean corpuscular hemoglobin (MCH). These six characteristics play an important role in the identification of TBPE and non-TBPE. Fever is a common clinical manifestation of tuberculous pleurisy (Kimura et al., 2002). Non-TBPE such as malignant tumors, transudate, blood disease, connective tissue disease often has no fever, but infectious pleural effusion can also be expressed as fever. Peripheral white blood cell count is increased in non-TBPE, may be related to pleural effusion of pneumonia in non-TBPE. This is the research results of Carrion-Valero and Perpina-Tordera (2001) and Neves et al. (2007), and the these two regard the peripheral white blood cell count as an important feature of distinguishing TBPE and non-TBPE models. The absolute value of eosinophils is increased in non-TBPE and may be related to lung cancer, lymphoma and multiple myeloma. In this study, the average age of TBPE was 41.85 years old, and age was selected as an important distinguishing feature, which may be related to China’s high prevalence of tuberculosis (Pan, 2012). In countries with high incidence of tuberculosis, tuberculous pleurisy is slightly in the younger, with an average age of 32–35 years (Valdes et al., 1998; Mihmanli et al., 2004; Ibrahim et al., 2005; Porcel et al., 2008). Whereas the average age of patients with tuberculous pleurisy in industrial countries is high. A study from the United States, from 1993 to 2003, showed 7,549 patients with tuberculous pleurisy had an average age of 45 years (Baumann et al., 2007). Monocytes are often increased in tuberculosis (Viloria et al., 2020) but are reduced in acute and chronic lymphocytic leukemia and all-bone marrow dysfunction diseases. Using the bGACO-SVM method, we selected the mean corpuscular hemoglobin as an important feature, with currently no similar study. The average amount of hemoglobin may be a potential predictor of TBPE.

In some diagnostic models, such as the artificial neural network model (ANN) of Seixas et al. (2013), the multivariate regression prediction model of Klimiuk et al. (2015) and the multi-factor prediction model of Shu et al. (2015) mentioned in the introduction section, need high requirements, or to detect some clinically not commonly used predictors. These predictors have not yet been routinely applied clinically, and the reagents are difficult to obtain and costly. In some underdeveloped regions and poor laboratory conditions, it’s difficult to carry out.

There are also some diagnostic models, such as Sales et al. (2009) which established two digital scoring models to distinguish TBPE and tumor pleural effusion. Diagnostic tuberculosis model 1, including characteristic variables ADA, globulin, and tumor cells with accuracy, sensitivity, specificity 97, 94.5, and 99.5%, respectively. Diagnostic tuberculosis model 2, including the characteristic variables ADA, globulin, and pleural effusion appearance with accuracy, sensitivity, specificity of 95.8, 95.5, and 96.1%, respectively. Porcel and Vives (2003) established a scoring system to identify TBPEs and tumor-related pleural effusions through multivariate analysis. Model 1 includes variable ADA, age, body temperature, pleural effusion RBC count, and the area under the ROC curve is 0.987, and sensitivity and specificity are 95 and 97%, respectively. Model 2 includes no tumor history, age, body temperature, chest water red cell count, pleural effusion LDH and serum LDH ratio, pleural effusion protein, and ROC curve of 0.982, with a sensitivity and specificity of 97 and 91%, respectively. Although these models are simple, diagnostic accuracy is high, but doesn’t include the various causes of pleural effusion in other patients, so only suitable for identifying cancerous pleural effusion and TBPE.

Our model has the advantages of simplicity, rapid prediction and low cost compared with the previous model, and the diagnostic accuracy is 90%. This model doesn’t require thoracocentesis; very suitable for diagnosing TBPE in patients with high difficulty obtaining pleural fluid such as less pleural fluid, a posterior scapular pleural effusion, encapsulated pleural effusion, etc. For non-invasive diagnosis, it is possible to avoid invasive pleural biopsy or thoracoscopy, which is more suitable for patients with severe pleural reaction or advanced age. The model is low cost and easy to detect, and it can be used in the economically underdeveloped areas and the high TB prevalence regions. It can be made as a phone or tablet app that requires no Internet connection to make it easier for clinicians to use it anywhere.

Our model also has some flaws. Sample size is small but the it can be expanded by continuous collection of pleural effusion cases, and the model can be optimized to improve the diagnostic accuracy. China is a highly epidemic country with tuberculosis, and the age of TB patients is significantly lower than that of tuberculosis low prevalence countries. Therefore, the model needs to be further validated in the low epidemic area of tuberculosis. We take into account the economic burden, didn’t join the blood gamma interferon release test or sputum NAA detection. These tests in economically underdeveloped areas are difficult to carry out. Including these detections or other biological indicators may improve the diagnostic accuracy.

## 7 Conclusion and future directions

In order to develop a study on the assisted diagnosis of TBPE, a high-performance classification prediction model, called bGACO-SVM, is proposed in this paper for the assisted diagnosis of TBPE from the perspective of swarm intelligence optimization and machine learning. bGACO-SVM consists of a classification prediction model combining a newly proposed swarm intelligence optimization algorithm GACO and a machine learning method SVM, where SVM is mainly used as a cost function of GACO to select the optimal subset of features. GACO, the core of bGACO-SVM, is an improved ant colony optimizer formed by introducing the grade-based search strategy into ACOR, which effectively compensates for the shortcomings of ACOR in terms of convergence performance and avoidance of local optima, and further enhances the performance of the bGACO-SVM model. In order to investigate the performance of GACO, this paper conducts basic algorithm comparison experiments and advanced variant algorithm comparison experiments using 30 benchmark functions in IEEE CEC2017 as the experimental basis. For the obtained experimental results, the Wilcoxon signed-rank test (García et al., 2010) and the Friedman test (Derrac et al., 2011) are mainly used to analyze the experimental results, which effectively prove that GACO has strong convergence ability and the ability to avoid falling into local optimum.

To investigate the classification prediction ability of bGACO-SVM for TBPE, we first validated it on the public dataset and then applied it to the TBPE prediction problem. During the experiments, bGACO-SVM was first compared with some similar algorithms on public datasets, and then, on the TBPE dataset, bGACO-SVM was compared not only with some similar algorithms, but also with five very classical machine learning methods. Five metrics, including accuracy, specificity, precision, MCC, and F-measure, are analyzed on the experimental simulation results from several perspectives, effectively demonstrating that bGACO-SVM has a strong classification prediction capability and can be successfully used for TBPE diagnosis prediction. However, the work in this paper also has some limitations. For example, by introducing grade-based search strategy into ACOR, the complexity of the algorithm is increased. However, these problems will be ready to be solved in the future by concurrency control of computers and future computer performance improvements. In conclusion, we have established a simple diagnostic model for predicting TBPE by the bGACO-SVM method of swarm intelligence algorithm, which achieves better diagnostic accuracy, sensitivity, and specificity by testing blood routine only. The low cost, fast diagnosis, non-invasive, and low equipment and technical requirements make it suitable for wide clinical application. Although the proposed GACO achieves very good performance on the benchmark functions, and the bGACO-SVM also shows some advantages on the classification prediction of TBPE, the introduction of the grade-based search strategy makes GACO have a large limitation in time complexity.

In the future, it is one of the most important works to overcome the time limitation of the proposed method by using high performance computing techniques. In addition, bGACO-SVM can be used for more disease diagnosis, and GACO can be applied to more optimization problems, such as: recommender system, image dehazing, medical image augmentation, and location-based services.

## Data availability statement

The original contributions presented in this study are included in the article/supplementary material, further inquiries can be directed to the corresponding author/s.

## Ethics statement

This research was carried out in accordance with the declaration of Helsinki and approved by the Medical Ethics Committee of The First Affiliated Hospital Wenzhou Medical University.

## Author contributions

CL, LH, JP, and GL: writing—original draft, writing—review and editing, software, visualization, and investigation. HC and XC: conceptualization, methodology, formal analysis, investigation, writing—review and editing, funding acquisition, and supervision. All authors contributed to the article and approved the submitted version.

## Footnotes

1
http://archive.ics.uci.edu/ml/index.php
